# Selenoprotein S maintains intestinal homeostasis in ulcerative colitis by inhibiting necroptosis of colonic epithelial cells through modulation of macrophage polarization

**DOI:** 10.7150/thno.97005

**Published:** 2024-09-09

**Authors:** Yujie Yao, Tong Xu, Xiaojing Li, Xu Shi, Hao Wu, Ziwei Zhang, Shiwen Xu

**Affiliations:** 1 College of Veterinary Medicine, Northeast Agricultural University, Harbin, 150030, PR China.; 2 School of Tropical Agriculture and Forestry, Hainan University, Haikou, 570228, PR China.; 3 College of Animal Science and Technology, Northeast Agricultural University, Harbin, 150030, PR China.; 4 Key Laboratory of the Provincial Education Department of Heilongjiang for Common Animal Disease Prevention and Treatment, College of Veterinary Medicine, Northeast Agricultural University, Harbin, 150030, PR China.

**Keywords:** Ulcerative Colitis, Macrophage Polarization, Selenoprotein S, Oxidative Stress, Necroptosis

## Abstract

**Rationale:** Macrophage polarization plays an important role in the inflammatory regulation of ulcerative colitis (UC). In this context, necroptosis is a type of cell death that regulates intestinal inflammation, and selenoprotein S (SelS) is a selenoprotein expressed in intestinal epithelial cells and macrophages that prevents intestinal inflammation. However, the underlying mechanisms of SelS in both cell types in regulating UC inflammatory responses remain unclear. Therefore, the direct effect of SelS deficiency on necroptosis in colonic epithelial cells (CECs) was investigated. In addition, whether SelS knockdown exacerbated intestinal inflammation by modulating macrophage polarization to promote necroptosis in CECs was assessed.

**Methods:** The UC model of SelS knockdown mice was established with 3.5% sodium dextran sulfate, and clinical indicators and colon injury were evaluated in the mice. Moreover, SelS knockdown macrophages and CECs cultured alone/cocultured were treated with IL-1β. The M1/M2 polarization, NF-κB/NLRP3 signaling pathway, oxidative stress, necroptosis, inflammatory cytokine, and tight junction indicators were analyzed. In addition, co-immunoprecipitation, liquid chromatography-mass spectrometry, laser confocal analysis, and molecular docking were performed to identify the interacting proteins of SelS. The GEO database was used to assess the correlation of SelS and its target proteins with macrophage polarization. The intervention effect of four selenium supplements on UC was also explored.

**Results: U**biquitin A-52 residue ribosomal protein fusion product 1 (Uba52) was identified as a potential interacting protein of SelS and SelS, Uba52, and yes-associated protein (YAP) was associated with macrophage polarization in the colon tissue of patients with UC. SelS deficiency in CECs directly induced reactive oxygen species (ROS) production, necroptosis, cytokine release, and tight junction disruption. SelS deficiency in macrophages inhibited YAP ubiquitination degradation by targeting Uba52, promoted M1 polarization, and activated the NF-κB/NLRP3 signaling pathway, thereby exacerbating ROS-triggered cascade damage in CECs. Finally, exogenous selenium supplementation could effectively alleviate colon injury in UC.

**Conclusion:** SelS is required for maintaining intestinal homeostasis and that its deletion enhances necroptosis in CECs, which is further exacerbated by promoting M1 macrophage polarization, and triggers more severe barrier dysfunction and inflammatory responses in UC.

## Introduction

Inflammatory bowel disease (IBD) is a chronic and relapsing inflammatory disease of the gastrointestinal tract, with Crohn's disease (CD) and ulcerative colitis (UC) as the primary disease patterns. Accumulated data from experimental models and clinical studies have indicated excessive immune cell infiltration and complex inflammatory networks as the main features of IBD 1. Macrophages are the gatekeepers of intestinal immune homeostasis, and studies have shown that macrophage polarization may initiate and resolve intestinal inflammation in patients with IBD and animal colitis models 2. In particular, M1 macrophages enhance tissue inflammation and exacerbate IBD damage, whereas M2 macrophages promote tissue repair and resolve inflammation to alleviate IBD symptoms 3. Recent studies have shown that the expression of yes-related protein (YAP), a downstream regulator of the Hippo signaling pathway, is altered during local intestinal inflammation, affecting the conversion of M1 and M2 macrophages. Indeed, YAP inhibits M2 polarization and aggravates intestinal inflammation. Meanwhile, conditional YAP knockout (KO) in macrophages has been shown to prevent M1 polarization and alleviate colitis in mice 4. However, the mechanism underlying the regulation of the M1/M2 switch and development of IBD remains unclear.

Intestinal epithelial cells (IECs) are essential for maintaining tissue homeostasis. However, excessive IECs death disrupts intestinal barrier integrity and leads to an inflammatory response in the lamina propria 5. Necroptosis, a novel form of cell death modality that regulates intestinal homeostasis and inflammation, involves the activation of the protein kinases RIPK1 and RIPK3, followed by the phosphorylation of the executioner molecule MLKL to induce cellular membrane rupture and release damage-associated molecular patterns, interleukin-1β (IL-1β) and other cytokines 6. Reactive oxygen species (ROS) have been suggested to contribute to necroptosis, although their origin and function in this process are not fully understood 7. The important role of necroptosis in certain inflammatory pathologies, such as IBD, sepsis, and neurodegenerative diseases, has been widely reported 8. Therefore, necroptosis inhibition is a potential therapeutic strategy for many diseases involving inflammation and cell death. However, the role of IEC necroptosis in the pathogenesis of IBD remains elusive.

Selenium is an essential trace mineral that participates in biological functions in the form of selenoproteins with selenocysteine (Sec), as the active center. Epidemiological investigations and clinical studies have identified reduced blood selenium level in patients with CD and UC 9, 10. Indeed, selenium deficiency promoted inflammatory responses and exacerbated intestinal damage in a mouse colitis model 11. Of note, the switch from the M1 to M2 phenotype of macrophages in the resolution phase of inflammation perhaps depends on the sufficient availability of selenium 12. Selenium supplementation to sodium dextran sulfate (DSS)-treated mice suppressed M1 markers and upregulated M2 markers in the colon tissue 13. However, the specific selenoproteins that affect macrophage polarization in UC remain unclear. The antioxidant and anti-inflammatory functions of selenoproteins suggest that they act as mediators to exert the beneficial effects of selenium in the gut, particularly via key roles played by them in host immune cells 14. A study on macrophage-specific Sec-tRNASec conditional KO mice revealed that selenoproteins in macrophages were critical for protecting against severe gastrointestinal injury and promoting efficient resolution 15. In addition, selenoprotein W (SelW) suppressed Th1 cell differentiation by promoting ROS scavenging in CD 16. Speckmann *et al.* indicated that selenoprotein S (SelS) was elevated in inflamed versus noninflamed ileal tissue of patients with CD 17. Furthermore, studies on the RAW264.7 cell line have shown that SelS regulated the release of cytokines from macrophages 18, indicating that SelS may be involved in regulating intestinal immune responses.

Although high SelS levels have been found in IECs and intestinal macrophages 17, little is currently known about its exact function in the intestine, particularly regarding the mechanism of SelS in regulating UC colonic epithelial damage caused by macrophage polarization. The present study revealed that SelS expression was increased specifically and severely in the colon of UC mice, whereas SelS KO promoted M1 polarization and exacerbated colonic epithelial cell (CEC) injury. Mechanistically, SelS deficiency in CECs induced ROS overproduction, necroptosis, inflammatory factor release, and tight junction dysfunction, and SelS deficiency in macrophages reduced the expression of the target protein ubiquitin A-52 residue ribosomal protein fusion product 1 (Uba52) to inhibit the ubiquitination degradation of YAP and promote the polarization of M1, which further exacerbated ROS burst-triggered injury in CECs. Of note, selenium supplementation promoted the expression of SelS and inhibited the severity and clinical symptoms of colitis in UC mice. These findings may enrich the biological functions of SelS and provide evidence that SelS regulates macrophage polarization to affect CEC injury, thereby simultaneously providing a theoretical basis for UC treatment and acting as a reference for comparative medicine.

## Materials

### Animals

All animal procedures were conducted in strict accordance with protocols approved by the Institutional Animal Care and Use Committee of Northeast Agricultural University (SRM-11). Wild-type (WT) C57BL/6J mice and SelS KO mice on C57BL/6J background were purchased from Cyagen Bioscienc Inc. (Jiangsu, China) and were bred under specific pathogen-free conditions with a 12-h light/12-h dark cycle, an ambient temperature of 22 ± 2 °C and humidity of 30-70%. Mice were co-housed with 4 mice per cage. The mice used at the beginning of the experiments were 8 weeks and weighed 25 ± 2 g. No gender bias was observed in both males and females.

### UC mouse model and selenium supplementation intervention

WT mice and SelS KO mice were administered 3.5% DSS (Macklin, Shanghai, China) in drinking water for 7 days. In the intervention experiment of selenium supplementation, sodium selenite (Na_2_SeO_3_), κ-selenocarrageenan (Se-Car), selenomethionine (SeMet), and nano-selenium (Nano-Se) were administered to WT mice by gavage at a dosage of 2 mg/kg selenium for 28 consecutive days, and then the mice drank distilled water containing 3.5% DSS for the last 7 days. The severity of colitis was scored by monitoring clinical disease activity through daily observations of the following parameters: weight loss (0 points = 1% weight loss, 1 points = 1-5% weight loss, 2 points = 6-10% weight loss, 3 points= 11-15% weight loss, and score 4 = >16% weight loss); stool dilution (0 points = normal and well-formed, 1 points = very soft and formed, 2 points = mild diarrhea, 3 points = moderate diarrhea, 4 points = severe diarrhea); and bleeding stool score (0 points = no occult blood, 1 points = slight occult blood, 2 points = moderate occult blood, 3 points = severe occult blood, 4 points = gross bloody stool). The Disease Activity Index (DAI) was the mean of the combined scores for weight loss, fecal consistency, and fecal occult blood. Mice were executed at the end of modeling; colon length was measured and tissues were stored at -80°C for backup.

### Histology and histopathological score

The distal colon segments were fixed in 4% paraformaldehyde for 24 h, the tissues were sequentially subjected to gradient ethanol dehydration, paraffin embedding, tissue sectioning, and hematoxylin and eosin (H&E) staining. Images were recorded using an optical microscope (Thermo Fisher Scientific, Waltham, MA, USA) to analyze morphology. The severity of colon injury was measured based on the histopathological score of inflammatory cell infiltration, extent, hyperplasia, goblet cell loss, ulceration, granulation tissue, and glandular rarefaction as previously described 19.

### Transmission electron microscopy (TEM) and ultrastructural score

The distal colon segments were fixed with 2.5% glutaraldehyde, then treated with 1% Osmium tetroxide, dehydrated with graded concentrations of ethanol, and embedded in a medium used for electron microscopy. Ultrathin sections of the colon were cut onto formvar-coated slot grids, and double-stained with 1% Uranyl acetate and 1% Lead citrate. Images were obtained with TEM (Hitachi, Tokyo, Japan). For facilitate presentation, the degree of damage to microvilli, epithelial cells, and tight junctions was scored according to the previous description 20.

### Cell culture and coculture

Mouse macrophage (J774.1), mouse CEC (MCEC), and mouse hepatocarcinoma cell (Hepa1-6) were cultured in complete Dulbecco's modified Eagle's medium (DMEM, Grand Island, New York, USA) supplemented with 10% fetal bovine serum (FBS, HyClone, Logan, USA) and 1% penicillin/streptomycin (Beyotime, Shanghai, China) at 37 ℃ in a humidified atmosphere with 5% CO_2_ strictly following aseptic protocol. The bone marrow-derived macrophages (BMDMs) were obtained from WT and SelS KO mice according to the previously reported protocol 21. In a coculture assay of MCEC and J774.1, MCECs were seeded on the bottom of 24-well transwell chamber plates, and J774.1 were seeded on top of 0.4-μm polycarbonate filter inserts in the transwell chamber plates (Corning, New York, USA). For loss-of-function assay, J774.1 were transfected with siNC (normal control) or siSelS. For inhibitor intervention assay, J774.1 were pretreated with YAP inhibitor Verteporfin (VP, 0.32 μM) for 30 min. Subsequently, both cells were cocultured with simultaneous exposure to 100 ng/mL IL-1β for 24 h. N-Acetylcysteine (NAC, 1 mM) was used to scavenge intracellular ROS by treating MCECs 30 min before coculture.

### siRNA transfection

J774.1 were transiently transfected with 50 nM of siRNA duplexes specific for SelS and Uba52 using Lipofectamine® RNAiMAX transfection reagent (Invitrogen, Carlsbad, CA, USA). BMDMs and MCECs were also transfected with siRNA for SelS. Cell transfected with scrambled siRNA and BLOCK-iT Alexa Fluor Red Fluorescent Oligo (Invitrogen, Carlsbad, CA, USA) were used as a negative and positive control, respectively. Following small RNA sequences were used. SelS: 5′-CAUGCAAGAAGGCAGAAGUUACAAA-3′, Uba52: 5′-CCGACTACAACATCCAGAA-3′. Cells were collected 48 hours after transfection for subsequent analysis.

### Co-immunoprecipitation (CoIP)

Whole-cell lysates were prepared by native lysis buffer with complete protease inhibitor cocktail and phosphatase inhibitors. 1000 mg protein was incubated with 2 mg specific antibodies at 4 ℃ overnight with constant rotation, followed by incubation with 50% Protein A/G Agarose beads (Absin Bioscience, Shanghai, China) for 2 h at 4 ℃. Subsequently, agarose beads were washed 3 times with lysis buffer and resuspended in an appropriate amount of SDS-PAGE loading buffer and boiling for 10 min. Samples from immunoprecipitation or cell lysates were analyzed by immunoblotting.

### Liquid chromatograph-mass spectrometry (LC-MS) analysis

Hepa1-6 cells were transfected with empty plasmid and Flag-SelS overexpression plasmid, respectively. After transfection for 48 h, cell lysates were purified with Anti-Flag M2 Affinity Gels (Sigma Aldrich, Missouri, USA) and eluted with Flag peptides. The LC-MS analysis was carried out by Sangon Biotech Co., Ltd. (Shanghai, China). Finally, we screened out the substrate proteins that could bind to SelS according to the score and the mass of detected differentially expressed proteins.

### Molecular docking

Zdock software was applied to predict the binding direction and affinity between SelS and Uba52 proteins. The FASTA sequence of SelS was downloaded from the UniProt database and uploaded to Swiss-Model for homology modeling. Simultaneously, the 3D structure for Uba52 (PDBID: 6JWI) was chosen from the PDB database. Then the protein structure files of both were uploaded to Zdock software for calculation, and the different docking solutions were ranked according to their energy scores. Pymol 2.3.0 software was applied to observe the interaction pattern of the first-ranked docking model.

### Detection of oxidative stress

Commercial test kits purchased from Nanjing Jiancheng Bioengineering Institute were employed to determine the contents of malonic dialdehyde (MDA) and glutathione (GSH), as well as the activities of glutathione peroxidase (GPX), catalase (CAT), total superoxide dismutase (T-SOD), and total antioxidant capacity (T-AOC). Specifically, fresh colon tissues were homogenized in cold physiological saline solution. The supernatants obtained after centrifugation were used to measure changes in these oxidative stress markers following the manufacturer's instructions. The 2′,7′-dichlorodihydrofluorescein diacetate (DCFH-DA) probe provided by Nanjing Jiancheng Bioengineering Institute was used to determine intracellular ROS accumulation in MCECs. After the cells were incubated in serum-free medium containing 10 μM DCFH-DA for 30 min, fluorescence images were obtained using a fluorescence microscope (Thermo Fisher Scientific, Waltham, MA, USA).

### Cytokine assay

Colonic homogenates were prepared using saline, and the contents of TNF-α, IL-1β, IFN-γ, IL-6, IL-10, and IL-17 in the collected supernatants were assayed using ELISA kits (Jingmei Biotechnology, Jiangsu, China) according to the manufacturer's instructions.

### Cell death assay

TdT-mediated dUTP nick-end labeling (TUNEL) staining was used to determine the apoptotic CECs in the colon tissue. 5 μm paraffin-embedded sections were incubated in a permeabilization solution and processed with the *In Situ* Apoptosis Detection Kit (Beyotime Biotechnology, Shanghai, China). *In vitro*, acridine orange/ethidium bromide (AO/EB) dual staining and flow cytometry were employed to analyze necroptotic MCECs. For AO/EB staining, cells were stained with a working solution containing 20 μL/mL AO and EB for 5 min, and the fluorescence signal was imaged under a fluorescence microscope. For flow cytometry, cells were labeled with Annexin V-FITC and propidine iodide (PI) according to the manufacturer's instructions (KeyGEN, Nanjing, China). Flowjo software provided a method for counting the rate of necroptosis.

### Total RNA isolation and quantitative real-time PCR (qRT-PCR)

Total RNA was isolated from colon tissues and treated cells with TRIzol^TM^ reagent (Invitrogen, Carlsbad, CA, USA). An amount of 2 mg total RNA was reverse-transcribed to cDNA by using a cDNA first strand synthesis kit (Bioer, Hangzhou, China). qRT-PCR was performed on a LineGene 9600 Plus (Bioer, Hangzhou, China) with SYBR Green Master Mix (Bioer, Hangzhou, China). The expression of individual genes was calculated with a 2-ΔΔCt method and normalized to the expression of ACTB. Gene-specific primer sequences are shown in Table S1.

### Protein isolations and immunoblots

Protein from colon tissues and treated cells were extracted using RIPA buffer containing protease inhibitor (PMSF, Beyotime Biotechnology, Shanghai, China) and phosphatase inhibitor (PhosSTOP, Roche). The protein concentration in the collected supernatants was determined by a BCA protein assay kit (Solarbio, Beijing, China). For western blot (WB) analysis, equal amounts of protein samples were separated by SDS-PAGE and transferred to nitrocellulose membranes (Pall Corporation, New York, USA), which were incubated with the primary antibodies for SelS (Sigma, HPA010025), Uba52 (Abcam, ab109227), YAP (ABclonal, A11264), p-YAP-S127 (ABclonal, AP0489), MST1 (ABclonal, A12963), p-MST1/2-T180/T183 (ABclonal, AP1094), LATS1 (ABclonal, A17992), p-LATS1-S909/LATS2-S872 (ABclonal, AP0904), NF-κB (Wanleibio, WL01980), p-NF-κB (Wanleibio, WL02169), NLRP3 (Wanleibio, WL02635), GSDMD-N (ABclonal, A24059), ASC (Wanleibio, WL02462), Caspase-1 (Wanleibio, WL02996), IL-1β (Wanleibio, WL00891), IL-18 (Wanleibio, WL01127), FADD (ABclonal, A19049), RIPK1 (ABclonal, A7414), p-RIPK1 (ABclonal, AP1230), RIPK3 (ABclonal, A5431), p-RIPK3 (ABclonal, AP1408), MLKL (ABclonal, A19685), p-MLKL (ABclonal, AP1173), TNF-α (Wanleibio, WL01581), IL-6 (Wanleibio, WL02841), IL-10 (Wanleibio, WL03088), Claudin1 (Wanleibio, WL03073), Occludin (Wanleibio, WL01996), ZO-1 (Wanleibio, WL03419), Ub (ABclonal, A18185), Flag (Sigma, F7425), HA (ABclonal, AE008), Myc (ABclonal, AE010), ACTB (ABclonal, AC038), then incubated with HRP-conjugated secondary antibodies and visualized by using an ECL kit (Biosharp, Hefei, China) in Azure Biosystem C300 imaging system (Thermo Fisher Scientific, Waltham, MA, USA). ACTB was applied to verify equal protein loading.

### Immunofluorescence

Colon sections and cells fixed with 4% PFA were permeabilized with 0.3% Triton X-100, then blocked with blocking buffer for 2 h at room temperature and incubated overnight with specific primary antibodies at 4 ℃. Subsequently, the samples were incubated with secondary antibody with fluorescent label. Images were captured using a fluorescence microscope and quantitatively analyzed with ImageJ.

### GEO data analysis

The GSE75214 and GSE59071 datasets were obtained through the GEO Database (https://www.ncbi.nlm.nih.gov/gds). Differential analysis was performed on the GSE75214 and GSE59071 datasets separately using the R package limma (version 3.4.6) to obtain differential genes between the comparison groups (UC and CD groups) and the control group. Next, Gene Set Variation Analysis (GSVA package of R http://www.bioconductor.org/) was used to explore the correlation between SelS, Uba52, YAP and the predefined, highly distinctive transcriptional profile of each immune cell type. The classical chemokines and surface markers of both M1 and M2 macrophages were also included. Twenty-six types of immune cells with corresponding gene signatures were utilized for analyses. Gene annotation and pathway enrichment analysis were performed by the Database for Annotation, Visualization, and Integrated Discovery (DAVID), and Kyoto Encyclopedia of Genes and Genomes (KEGG, http:// www.kegg.jp/kegg/pathway.html).

### Quantification and statistical analysis

Statistical analysis was performed using GraphPad Prism 9 software. All data are given as the mean ± s.e.m of at least three biological replicates. Two groups were compared by unpaired *t*-test if data were normally distributed and otherwise by Mann-Whitney U test. Comparisons between multiple groups were done by one-way or two-way ANOVA with a Bonferroni multiple comparison post-test. Statistical significance was set at *p* < 0.05. *In vitro* significance was indicated as *p < 0.05, *p < 0.01, and ***p < 0.001. *In vivo* significance was indicated by different letters, with the same letter representing no difference significance and different letters representing difference significance.

## Results

### SelS ablation aggravates colon injury in UC mice

**Figure 1:**
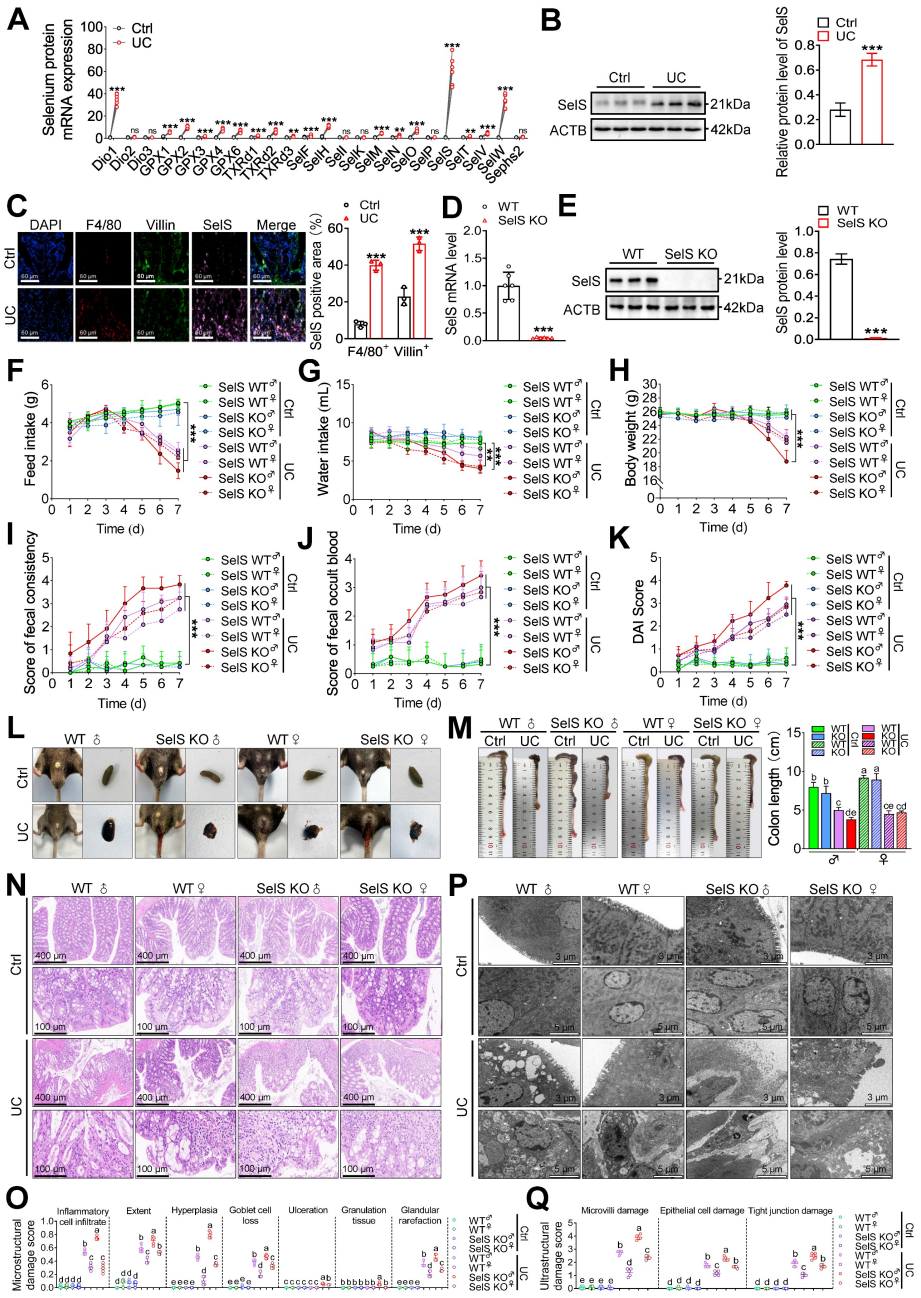
** SelS deletion aggravates colon injury in UC mice. (A)** Expression profile of 25 selenoproteins in the colon of WT mice with UC, n = 6. **(B)** SelS protein levels in the colon of WT mice with UC, n = 3. **(C)** Immunofluorescence staining and quantitative analysis of F4/80, Villin, and SelS in the colon of WT mice with UC, n = 3. Scale bar, 60 μm. **(D)** SelS mRNA expression in the colon of WT mice and SelS KO mice, n = 6. **(E)** SelS protein level in the colon of WT mice and SelS KO mice, n = 3. **(F-K)** Clinical indicators of WT and SelS KO mice in Ctrl and UC groups, n = 6. **(F)** Feed intake. **(G)** Water intake. **(H)** Body weight. **(I)** Scores of fecal consistency. **(J)** Scores of fecal occult blood. **(K)** DAI score. **(L)** Perianal area and feces appearance of WT and SelS KO mice in Ctrl and UC groups. **(M)** Colon length of WT and SelS KO mice in Ctrl and UC groups, n = 6. Different lowercase letters indicate significant differences between groups. **(N)** Representative H&E slides of distal colon sections of WT and SelS KO mice in Ctrl and UC groups, n = 6. Scale bar, 400 μm and 100 μm. **(O)** The microstructural damage score of the colon, n = 6. Different lowercase letters indicate significant differences between groups. **(P)** Representative images of TEM detection in Ctrl and UC groups for the colon of WT and SelS KO mice, n = 6. Scale bar, 3 μm and 5 μm. **(Q)** The ultrastructural damage score of the colon, n = 6. Different lowercase letters indicate significant differences between groups.

To investigate the role of SelS in colon injury in UC mice, the mRNA expression of 25 selenoproteins, including SelS, was assessed in the colon tissue of UC mice. Compared with the control group, SelS exhibited the highest expression abundance (81.94-fold) (Figure 1A). Its protein expression was significantly increased in the cytoplasm of macrophages (F4/80^+^) and CECs (Villin^+^) in the UC group (Figure 1B-C). To determine whether SelS plays a positive or negative role in UC, a genetic SelS KO mouse strain using CRISPR/CAS9-mediated genome engineering was established (Figure 1D-E). Under normal conditions, SelS KO mice displayed similar health status to WT mice. However, in the UC model mice, the feed intake, water intake, and body weight were significantly lower (Figure 1F-H); fecal consistency, fecal occult blood, and DAI scores were significantly increased (Figure 1I-L); and the colon segment from the cecum to rectum was shortened (Figure 1M). In addition, SelS KO mice exhibited more pronounced changes than WT mice. H&E staining revealed that inflammatory infiltration, infiltration extent, hyperplasia, goblet cell loss, ulceration, and glandular rarefaction in SelS KO mice with UC were more severe than those in WT mice with UC (Figure 1N-O). TEM showed that the damage to the microvillus, epithelial cell, and tight junction in SelS KO mice was more pronounced than that in WT mice with UC (Figure 1P-Q), indicating that SelS ablation exacerbates colonic histopathological and microstructural damage in UC. The role of sex in colon injury was also assessed. Of note, males exhibited more severe clinical symptoms and colonic lesions than females in both WT and SelS KO mice. Considering that hormone secretion in females may interfere with inflammatory response and thus inhibit UC development in clinical case analysis, male mice were selected for subsequent experiments. Overall, these findings suggest that SelS exhibits a positive regulatory effect on colon injury and that its inactivation worsens UC.

### SelS ablation drives M1 polarization in UC colon by upregulating macrophage YAP expression

**Figure 2:**
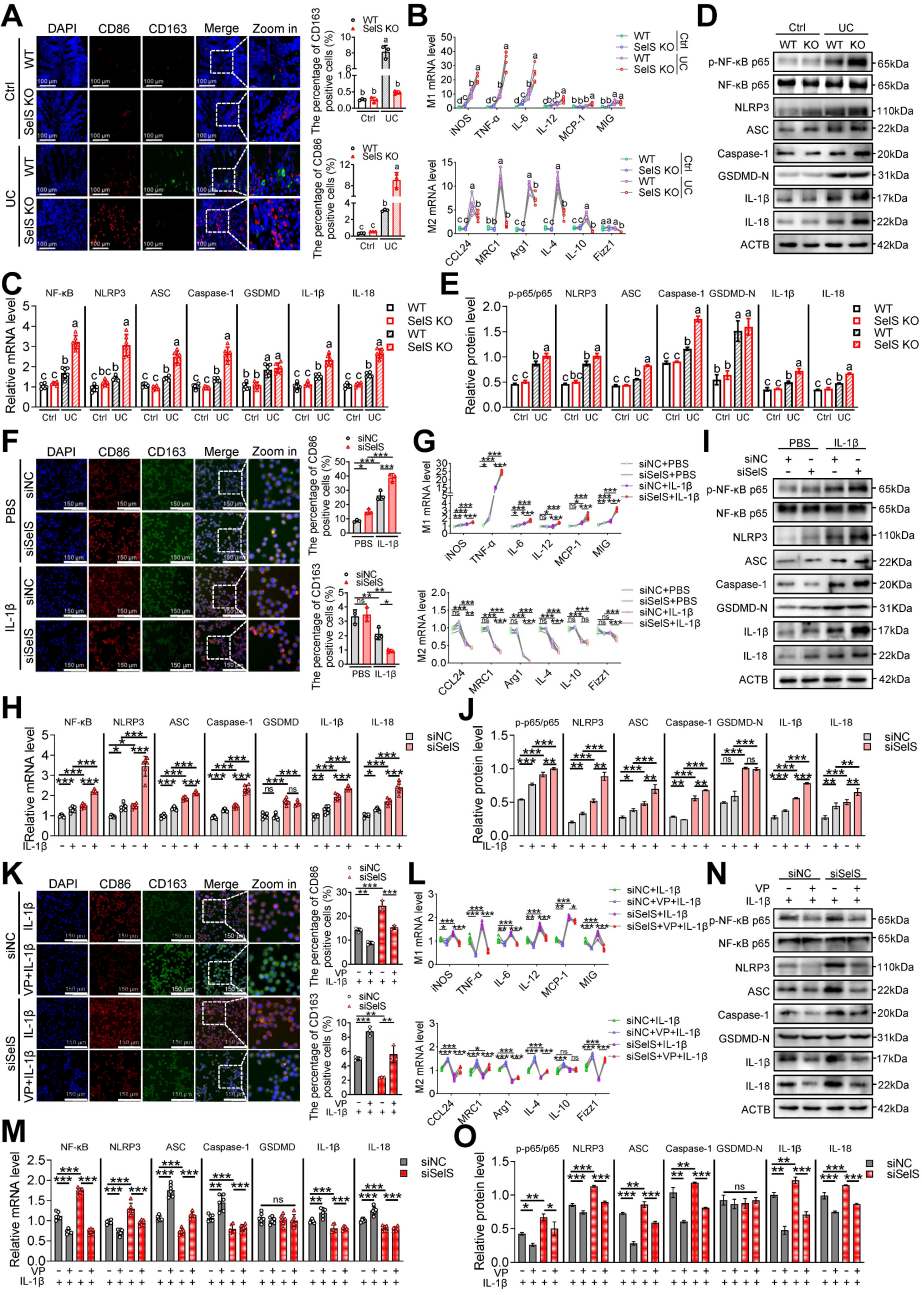
** SelS ablation promotes YAP-mediated M1 polarization and NF-κB/NLRP3 pathway activation. (A)** Immunofluorescence staining and quantitative analysis of CD86 and CD163 in the colon of WT and SelS KO mice in Ctrl and UC groups, n = 3. Scale bar, 100 μm. Different lowercase letters indicate significant differences between groups. **(B)** mRNA expressions related to M1 and M2 in the colon of WT and SelS KO mice in Ctrl and UC groups, n = 6. Different lowercase letters indicate significant differences between groups. **(C)** mRNA expressions related to the NF-κB/NLRP3 pathway and GSDMD in the colon of WT and SelS KO mice in Ctrl and UC groups, n = 6. Different lowercase letters indicate significant differences between groups.** (D-E)** Protein levels related to the NF-κB/NLRP3 pathway and GSDMD in the colon of WT and SelS KO mice in Ctrl and UC groups, n = 3. Different lowercase letters indicate significant differences between groups. **(F-J)** J774.1 transfected with siNC or siSelS were stimulated with PBS or IL-1β (100 ng/mL) for 6 h.** (F)** Immunofluorescence staining and quantitative analysis of CD86 and CD163 in J774.1, n = 3. Scale bar, 150 μm. **(G)** mRNA expressions related to M1 and M2 in J774.1, n = 6. **(H)** mRNA expressions related to the NF-κB/NLRP3 pathway and GSDMD in J774.1, n = 6. **(I-J)** Protein levels related to the NF-κB/NLRP3 pathway and GSDMD in J774.1, n = 3. **(K-O)** J774.1 transfected with siNC or siSelS were pretreated with or without VP (0.32 μM) for 30 min before PBS or IL-1β (100 ng/mL) stimulation for 6 h. **(K)** Immunofluorescence staining and quantitative analysis of CD86 and CD163 in J774.1, n = 3. Scale bar, 150 μm.** (L)** mRNA expressions related to M1 and M2 in J774.1, n = 6. **(M)** mRNA expressions related to the NF-κB/NLRP3 pathway and GSDMD in J774.1, n = 6.** (N-O)** Protein levels related to the NF-κB/NLRP3 pathway and GSDMD in J774.1, n = 3.

To elucidate the pathological significance of YAP in the SelS modulation of macrophage activity and skewing in the UC colon, the infiltration and polarization of macrophages in the colon tissue were determined. The results showed that SelS KO had no effect on the rising number of F4/80-labeled macrophages in the UC colon (Figure S1) but that it promoted and inhibited the increase of M1 and M2 macrophages, respectively (Figure 2A). Likewise, the mRNA expression of M1 and M2 markers showed the same pattern in the colon of SelS KO mice with UC (Figure 2B). In addition, SelS KO enhanced the activation of the NF-κB/NLRP3 pathway but did not alter GSDMD expression (Figure 2C-E). Because IL-1β is an important inflammatory factor involved in the inflammatory response during UC progression and significantly increased in the colon tissue of SelS KO mice with UC (Figure 7A), the effect of SelS on macrophage polarization was analyzed by treating SelS-knockdown macrophages with IL-1β *in vitro*. In agreement with the *in vivo* results, the number of M1 macrophages and expression of associated genes at the mRNA level were significantly augmented in SelS-deficient macrophages, and the opposite was observed in the M2 polarization assay (Figure 2F-G). Moreover, the silencing of SelS upregulated the expression of genes related to the NF-κB/NLRP3 pathway without affecting GSDMD expression in J774.1 (Figure 2H-J), consistent with the results observed in colonic macrophages (Figure S7C) and suggesting that SelS deletion has no effect on macrophage pyroptosis. Of note, exposing IL-1β-treated siSelS J774.1 to the YAP inhibitor VP effectively reversed the polarization state of macrophages (Figure 2K-L) and activation of the NF-κB/NLRP3 signaling pathway, again without affecting GSDMD expression (Figure 2M-O). The same changes were observed in M1/M2 polarization, NF-κB/NLRP3 pathway, and GSDMD in BMDMs (Figure S2). Taken together, SelS deficiency exacerbated M1 polarization in the UC colon by promoting YAP expression in macrophages.

### SelS-targeted Uba52 promotes ubiquitination degradation of macrophage YAP protein in a proteasome-dependent manner instead of the Hippo pathway

To clarify the regulatory effect of SelS on YAP, Hippo signaling pathway-related indicators were first examined. The results revealed that *in vivo*, the mRNA expression and phosphorylated protein levels of MST1, LATS1, and YAP were unchanged in the colon tissue of UC and SelS KO mice (Figure S3A-C). However, SelS KO significantly increased the total protein level of YAP (Figure S3B-C), suggesting that the negative regulatory effect of SelS on YAP protein expression in the UC colon is not regulated at the transcriptional level. In addition, the effect was not dependent on the regulation of a series of kinase cascades upstream of the Hippo signaling pathway. Meanwhile, *in vitro*, the treatment of J774.1 with IL-1β did not affect the mRNA expression and phosphorylated protein levels of MST1, LATS1, and YAP (Figure S3D-I). However, the treatment increased the total protein level of YAP in a time- and dose-dependent manner, except at 8 h (Figure S3E-F and H-I), suggesting that IL-1β promoted macrophage YAP protein level at the post-translational stage, independent of the traditional Hippo pathway.

**Figure 3:**
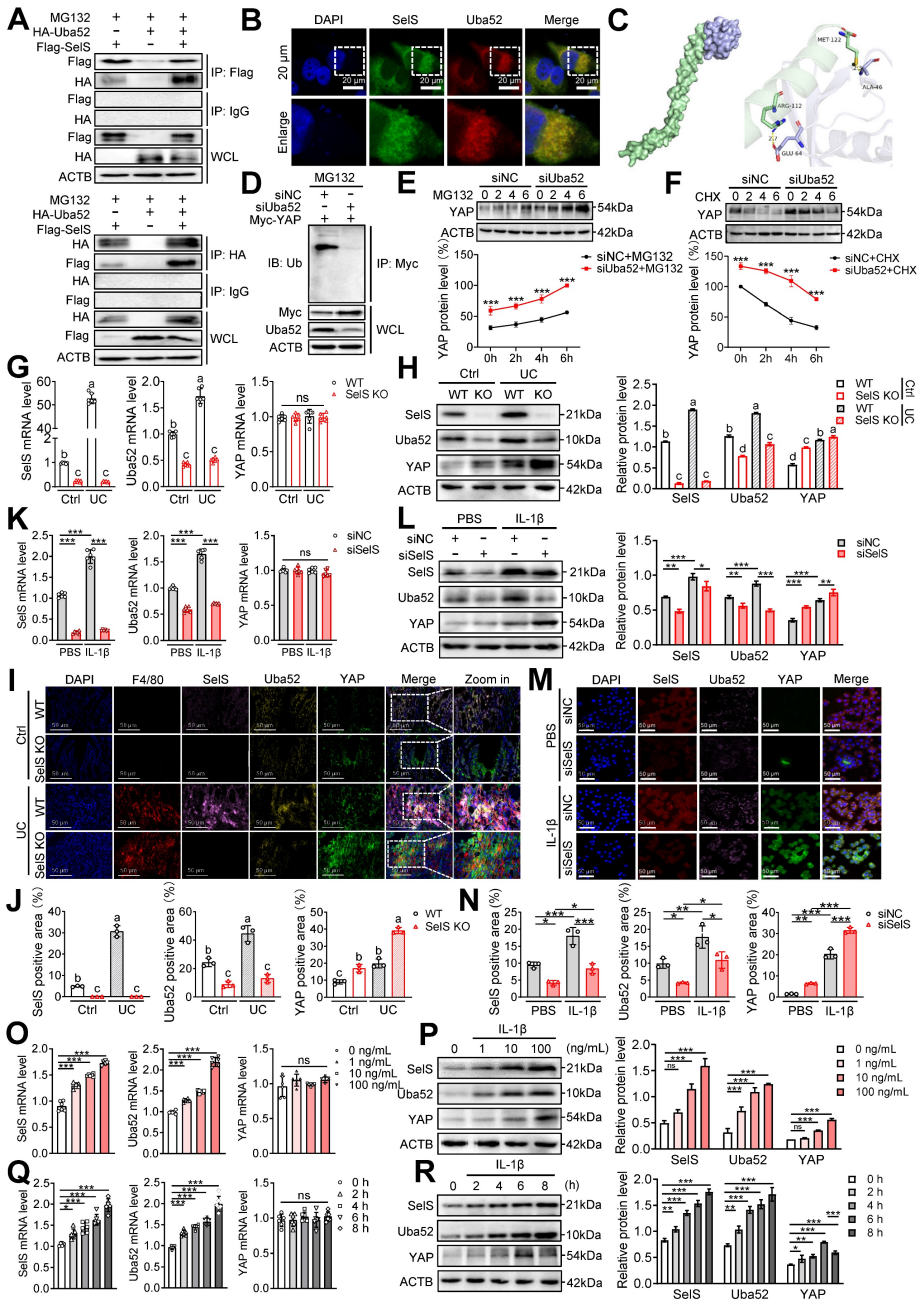
** SelS negatively regulates the stability of macrophage YAP by promoting the Uba52-dependent proteasome degradation pathway. (A)** The IB of Flag or HA was followed by immunoprecipitation (IP) with anti-HA antibody or anti-Flag antibody in whole-cell lysates (WCL) of J774.1, n = 3. **(B)** Immunofluorescence co-localization of SelS and Uba52 in J774.1, n = 3. Scale bar, 20 μm.** (C)** Molecular docking pattern diagram of SelS and Uba52. **(D)** J774.1 were cotransfected with Myc-YAP in the presence or absence of siUba52 and then treated with MG132 (10 mM) for 6 h. The cell lysates were subjected to IP with anti-Myc beads and immunoblotted with the ubiquitination antibody, n = 3. **(E)** J774.1 were pretreated with siNC or siUba52 and then incubated with MG132 (10 mM) for indicated time points. WB analysis of endogenous YAP, n = 3. **(F)** J774.1 were pretreated with siNC or siUba52. Subsequently, stimulated with IL-1β for 6 h before being incubated with CHX (50 mg/mL) for indicated time points. WB analysis of endogenous YAP, n = 3. **(G-J)** WT and SelS KO mice were treated with or without 3.5% DSS for 7 d. **(G-H)** mRNA (n = 6) and protein (n = 3) expression of SelS, Uba52 and YAP in the colon. Different lowercase letters indicate significant differences between groups. **(I-J)** Immunofluorescence staining and quantitative analysis of SelS, Uba52, and YAP in the colon. n = 3. Scale bar, 50 μm. Different lowercase letters indicate significant differences between groups.** (K-N)** J774.1 transfected with siNC or siSelS were treated with PBS or IL-1β (100 ng/mL) for 6 h. **(K-L)** mRNA (n = 6) and protein (n = 3) expression of SelS, Uba52, and YAP in J774.1. **(M-N)** Immunofluorescence staining and quantitative analysis of SelS, Uba52, and YAP in J774.1. n = 3. Scale bar, 50 μm. **(O-P)** J774.1 were stimulated with indicated concentrations of IL-1β for 6 h. mRNA (n = 6) and protein (n = 3) expression of SelS, Uba52, and YAP. **(Q-R)** J774.1 were treated with IL-1β (100 ng/mL) for the indicated times. mRNA (n = 6) and protein (n = 3) expression of SelS, Uba52, and YAP.

To investigate how SelS regulates YAP expression in macrophages during UC development, proteins interacting with SelS were determined using CoIP and LC-MS analysis in Hepa1-6 and the obtained 372 differentially expressed proteins were analyzed using bioinformatics (Figure S4). CoIP assay demonstrated that SelS physically associated with Uba52 in cotransfected J774.1 (Figure 3A). In addition, immunofluorescence results indicated that SelS and Uba52 spatially colocalized in J774.1 (Figure 3B). Besides, molecular docking analysis displayed the binding sites for SelS and Uba52 (Figure 3C).

These results confirmed the existence of interaction between Uba52 and SelS. Meanwhile, compared with siNC J774.1, siUba52 J774.1 showed profoundly less ubiquitination of YAP (Figure 3D), suggesting that Uba52 promotes the ubiquitination degradation of YAP. Next, the protein stability of YAP was evaluated with the proteasome inhibitor MG132 and the protein synthesis inhibitor cycloheximide (CHX). The results revealed that endogenous YAP protein accumulated in the presence of MG132 from 2 h and further increased at 4 and 6 h after treatment and that the protein expression of YAP in the siUba52 group was higher than that in the siNC group at each time point (Figure 3E). The turnover rate of YAP protein detected with CHX in IL-1β-treated J774.1 showed that the YAP protein level decreased gradually with the increase in time point and the decline rate in the siUba52 group was lower than that of the siNC group (Figure 3F). These results suggest that YAP is a short-lived protein in macrophages that is rapidly degraded via the proteasomal pathway dependent on Uba52. SelS and Uba52 are known to interact while Uba52 negatively regulates YAP protein expression by promoting its ubiquitinated degradation. However, the study results revealed YAP protein levels were always upregulated with the increasing mRNA and protein levels of SelS and Uba52 in UC mice (Figure 3G-H and I-J) and IL-1β-treated J774.1 (Figure 3K-L and M-N), suggesting that other pathways regulate YAP expression. Furthermore, the protein level of YAP in SelS KO mice and siSelS macrophages was upregulated with the decreasing mRNA and protein levels of SelS and Uba52 (Figure 3G-N), providing strong evidence that SelS negatively regulates YAP protein expression. Additional experiments revealed that IL-1β increased the protein expression level of SelS, Uba52, and YAP in J774.1 in a dose-dependent manner (Figure 3O-P). However, with prolonged IL-1β stimulation, YAP protein levels reached a maximum at 6 h but significantly decreased at the peak of SelS and Uba52 protein expression (8 h) (Figure 3Q-R), indicating that SelS negatively regulates YAP stability in macrophages by targeting Uba52 to promote its proteasome-dependent degradation.

### Correlation of SelS, Uba52, and YAP with immune cell types in individuals with IBD revealed via GEO database mining

**Figure 4:**
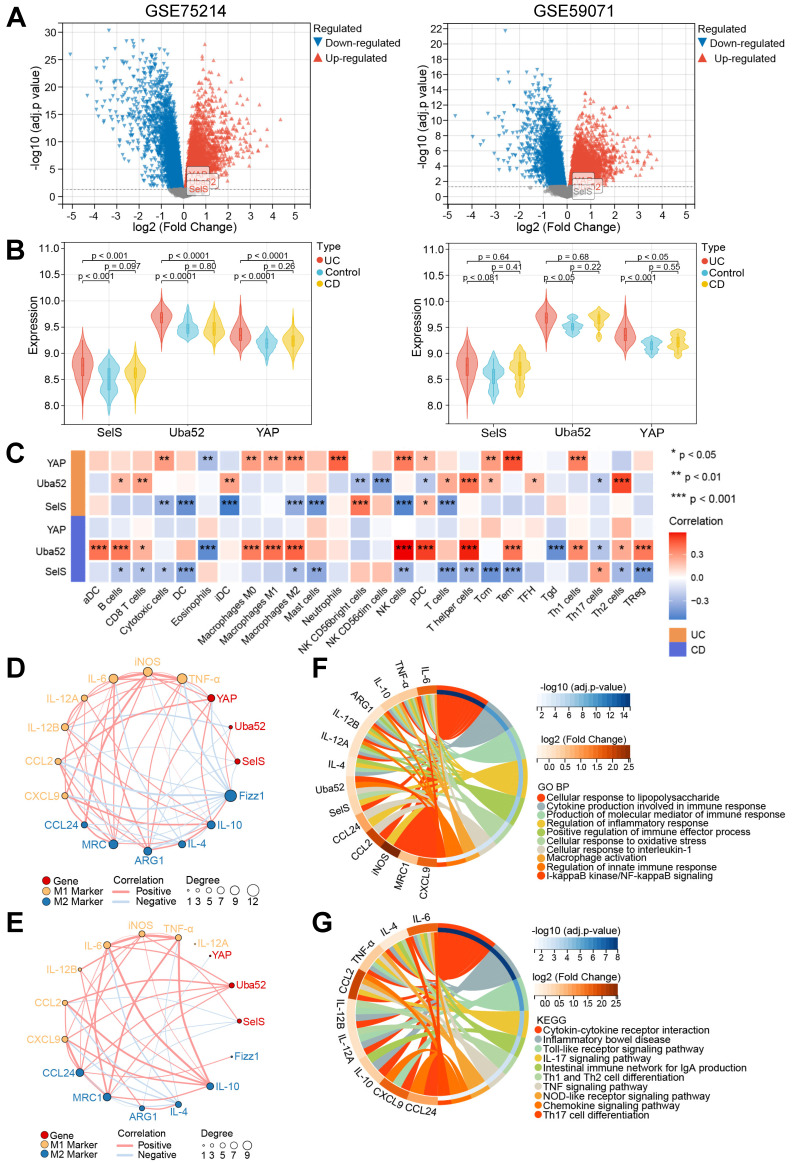
** Relationship between SelS, Uba52, and YAP and macrophage polarization in the colon of IBD patients mined by GEO data. (A)** Volcano plot depicting DEGs between healthy individuals and IBD patients in the GSE75214 and GSE59071 datasets. **(B)** Violin plots depicting differences in SelS, Uba52, and YAP expression between healthy individuals and IBD patients in the GSE75214 and GSE59071 datasets. **(C)** Heatmap indicates the correlation of SelS, Uba52, and YAP with 26 immune cell subpopulations in the TCGA dataset. **(D)** Correlation between SelS, Uba52, YAP, and M1/M2 macrophage polarization markers in UC patients in the GSE75214 dataset. **(E)** Correlation between SelS, Uba52, YAP, and M1/M2 macrophage polarization markers in CD patients in the GSE75214 dataset. **(F)** Biological processes (BP) analyzed by GO functional annotation of gene sets consisting of SelS, Uba52, YAP, and M1/M2 biomarkers. **(G)** KEGG enrichment analysis of gene sets composed of SelS, Uba52, YAP, and M1/M2 biomarkers.

A total of 194 and 116 sample data were obtained from the GSE75214 and GSE59071 datasets, respectively. The GSE75214 dataset contains 22 healthy control, 97 UC, and 75 CD samples. Differential expression analysis in this dataset yielded 979 DEGs, among which 518 were upregulated and 416 downregulated. Meanwhile, the GSE59071 dataset includes data from 11 healthy control, 97 UC, and 8 CD samples, and differential expression analysis in this dataset identified 911 DEGs, of which 598 were upregulated and 313 downregulated (Figure 4A). Next, the differential expression of SelS, Uba52, and YAP in patients with IBD and healthy controls was assessed. As shown in Figure 4B, the expression level of Uba52 and YAP in patients with UC was significantly higher than that in healthy individuals in both the GSE75214 and GSE59071 datasets. However, SelS was significantly increased only in the GSE75214 dataset. Meanwhile, no differences in the expression level of the above three genes were observed between patients with CD and healthy individuals in either dataset.

To investigate the possible impact of the three genes on the turbulence of the colonic immune microenvironment during IBD onset, the correlation among SelS, Uba52, YAP, and 26 types of immune cells, particularly macrophages, was explored using Pearson correlation analysis. In patients with UC SelS was negatively correlated with M2 macrophages (cor = -0.338) and Uba52 was positively correlated with Th2 cells (cor = 0.509). Besides, YAP was positively associated with 10 immune cell types, including M1 (cor = 0.275) and M2 macrophages (cor = 0.333). In patients with CD, SelS was negatively correlated with various immune cell types, including M2 macrophages (cor = -0.268), and Uba52 was positively correlated with both M2 (cor = 0.442) and M1 macrophages (cor = 0.441). However, YAP was not correlated with the above 26 types of immune cells (Figure 4C). Therefore, it was hypothesized that SelS, Uba52, and YAP exhibit different response patterns to macrophage polarization in individuals with CD and UC.

Next, the association of SelS, Uba52, and YAP with the biomarkers of M1 and M2 macrophages in IBD samples was analyzed. Significant correlation differences were observed in patients with UC and CD, with SelS and YAP showing negative and positive correlation with the M1 markers, respectively, in patients with UC (Figure 4D) and Uba52 exhibiting positive correlation with both M1 and M2 markers in patients with CD (Figure 4E). To assess the function of SelS, Uba52, and YAP in modulating macrophage polarization, these three genes as well as M1 and M2 biomarkers were used as gene ensembles to identify potential biological pathways. For this, Gene Ontology (GO) analysis revealed multiple biological processes. These included the production of cytokines involved in immune responses, production of the molecular mediators of immune responses, regulation of inflammatory responses, positive regulation of immune effector processes, cellular responses to oxidative stress, activation of macrophages, regulation of innate immune responses, and IκB kinase/NF-κB signaling (Figure 4F). In addition, KEGG analysis revealed multiple pathways, including cytokine-cytokine receptor interactions, inflammatory bowel disease, Toll-like receptor signaling pathway, Th1 and Th2 cell differentiation, TNF-α signaling pathway, NOD-like receptor signaling pathway, and chemokine signaling (Figure 4G).

### Inhibition of YAP attenuates SelS ablation-induced M1 polarization, resulting in oxidative stress amelioration in CECs

**Figure 5:**
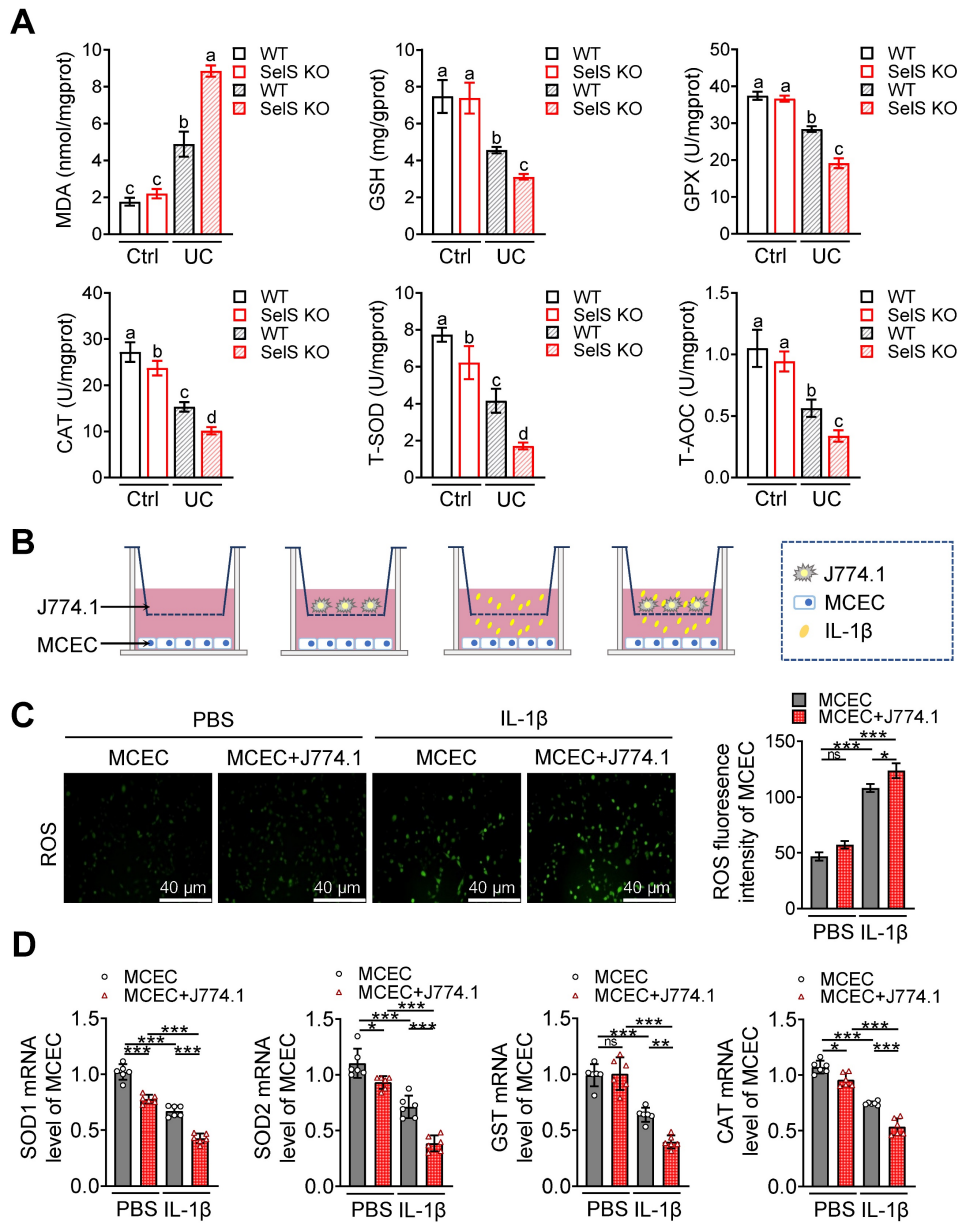
** SelS deletion exacerbates oxidative stress in the colon of UC mice involves M1 macrophages inducing ROS overproduction in CECs. (A)** Levels of pro-oxidant indicators (MDA) and antioxidant markers (GSH, GPX, CAT, T-SOD, and T-AOC) in the colon of WT and SelS KO mice in Ctrl and UC groups, n = 6. Different lowercase letters indicate significant differences between groups.** (B-D)** MCECs were cocultured with J774.1 under IL-1β (100 ng/mL) stimulation. **(B)** Coculture pattern diagram of MCECs and J774.1. **(C)** Representative images and quantitative analysis of ROS levels in MCECs, n = 3. Scale bar, 40 μm. **(D)** mRNA expression of antioxidase in MCECs, n = 6.

Pathological damage in UC is generally accompanied by oxidative stress in the colonic epithelium. Thus, whether SelS deficiency directly affects the redox status of CECs was investigated. Given that macrophages create a specific microenvironment for oxidative damage in various cells by employing a pro-inflammatory profile, whether SelS insufficiency changes the redox environment in CECs by promoting M1 polarization was also assessed. As shown in Figure 5A, the MDA content was increased, but the GSH content and GPX, CAT, T-SOD, and T-AOC activities in WT mice were diminished in the UC model. SelS KO enhanced these changes compared with WT, indicating that SelS deficiency aggravates oxidative stress *in vivo*. Meanwhile, higher ROS levels and lower SOD1, SOD2, GST, and CAT mRNA expression were observed in IL-1β-treated MCECs of siSelS than that with siNC treatment (Figure S5A-B), suggesting that SelS deletion in CECs directly induces oxidative stress. In addition, ROS produced by MCECs alone or in combination with macrophages (J774.1) exposed to IL-1β was significantly increase (Figure 5B-C), accompanied by reduced SOD1, SOD2, GST, and CAT mRNA expressions in MCECs (Figure 5D). Moreover, ROS level was abnormally increased and the aforementioned antioxidant gene expression was decreased in MCECs cocultured with the J774.1 of siSelS compared with siNC. However, this phenomenon was reversed by VP, which inhibited YAP expression in J774.1 (Figure S6A-C), suggesting that SelS deficiency in macrophages promotes YAP-dependent M1 polarization and leads to oxidative stress in CECs. Next, whether the redox imbalance of CECs is ameliorated by ROS-scavenging NAC was assessed. As shown in Figure S6D-F, abnormal ROS level and antioxidant gene expression in MCECs cocultured with J774.1 were restored to baseline under NAC treatment, indicating the beneficial effect of NAC in attenuating oxidative stress.

### SelS ablation drives M1 polarization and exacerbates oxidative stress-mediated necroptosis in CECs

**Figure 6:**
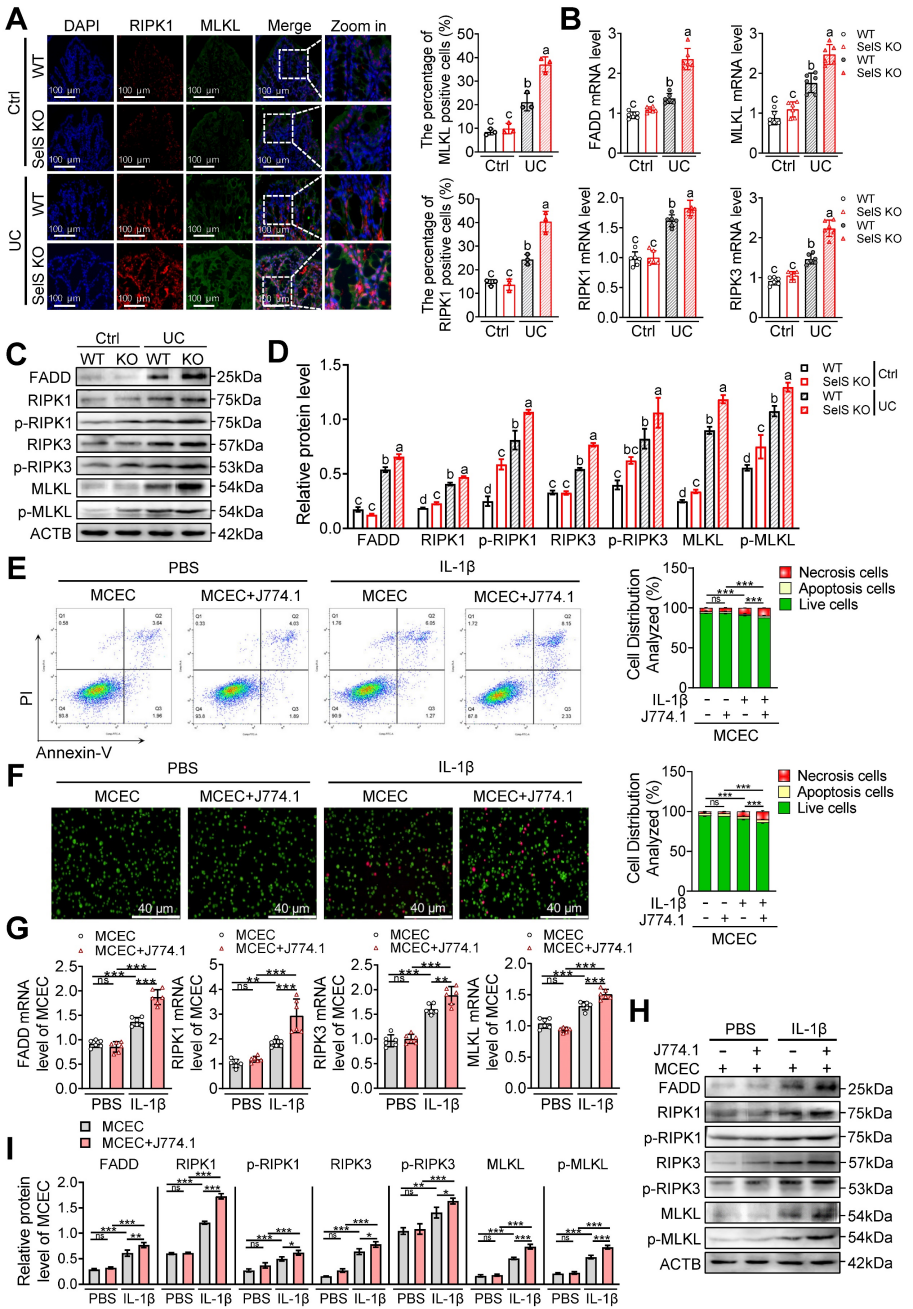
** SelS deletion promotes necroptosis in the colon of UC mice involves M1 macrophages inducing oxidative stress in CECs. (A)** Immunofluorescence staining and quantitative analysis of RIPK1 and MLKL in the colon of WT and SelS KO mice in Ctrl and UC groups, n = 3. Scale bar, 100 μm. Different lowercase letters indicate significant differences between groups.** (B)** mRNA expressions related to necroptosis in the colon of WT and SelS KO mice in Ctrl and UC groups, n = 6. Different lowercase letters indicate significant differences between groups. **(C-D)** Protein levels related to necroptosis in the colon of WT and SelS KO mice in Ctrl and UC groups, n = 3. Different lowercase letters indicate significant differences between groups. **(E-I)** MCECs were cocultured with J774.1 under IL-1β (100 ng/mL) stimulation. **(E)** Flow cytometry detection and quantification analysis for FITC/PI staining in MCECs, n = 3. **(F)** AO/EB staining and quantitative analysis in MCECs, n = 3. Scale bar, 40 μm. **(G)** mRNA expressions related to necroptosis in MCECs, n = 6. **(H-I)** Protein levels related to necroptosis in MCECs, n = 3.

It is widely accepted that only the strict control of the proliferation and death of IECs can ensure the integrity of the intestinal structure and maintenance of an effective intestinal barrier. Apoptosis, pyroptosis, and necroptosis are programmed cell death forms closely related to UC. To unravel the underlying mechanism through which SelS deficiency leads to worsening colon injury, Ki67, TUNEL, and GSDMD immunofluorescence staining were performed using the colon tissue of WT and SelS KO mice. The results revealed that despite the colon tissue of the UC group exhibiting fewer Ki67-positive cells and more TUNEL- and GSDMD-positive cells compared with the Ctrl group, no significant differences were observed between WT and SelS KO mice in the UC group (Figure S7A-C), suggesting that SelS deletion does not aggravate colon injury by affecting proliferation, apoptosis, and pyroptosis. Besides, immunofluorescence staining revealed an increased expression of the necroptosis biomarkers RIPK1 and MLKL in the UC group compared with the Ctrl group. Of note, the relative fluorescence intensity of RIPK1 and MLKL in the colon tissue of SelS KO mice was dramatically higher than that in WT mice within the UC group (Figure 6A). Furthermore, the results of the quantitative analysis of necroptosis-related mRNA, total protein, and phosphorylated protein were consistent (Figure 6B-D). Meanwhile, SelS knockdown upregulated the necroptosis rate (Figure S8A-D) and the gene expression level of the necroptosis markers (Figure. S8E-G) of MCECs treated with IL-1β. Therefore, these results indicate that the occurrence of necroptosis is critical for SelS deficiency to aggravate colon injury. Additionally, coculture with IL-1β-induced J774.1 upregulated the necroptosis rate (Figure 6E-F) and the gene expression level of relevant necroptosis markers (Figure 6G-I) in MCECs. More importantly, a necroptosis was substantially increased in MCECs cocultured with SelS-silenced J774.1 (Figure S9A-D). However, these effects were corrected with the VP intervention of J774.1 (Figure S9A-D) and NAC treatment of MCECs (Figure S9H-K), reversing the expression patterns of both mRNA and proteins (Figure S9E-G and L-N). Thus, these results suggest that SelS deficiency induces M1 polarization by upregulating macrophage YAP expression, thereby promoting oxidative stress in CECs and ultimately exacerbating necroptosis.

### SelS ablation exacerbates inflammatory response depending on oxidative stress in CECs induced by M1 polarization

**Figure 7:**
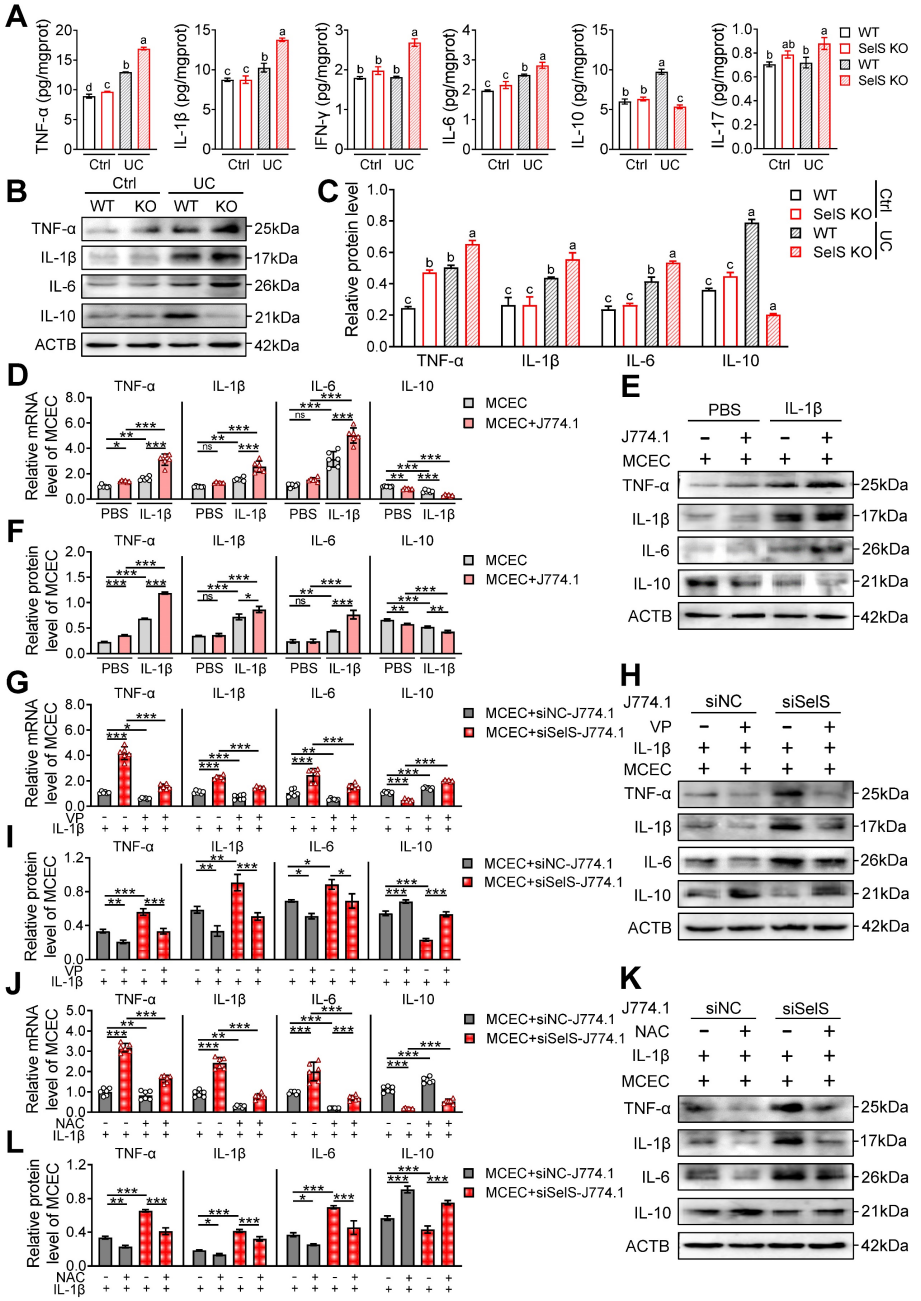
** SelS deletion amplifies inflammatory response relies on M1 polarization-induced oxidative stress in CECs. (A)** Inflammatory cytokine levels in the colon of WT and SelS KO mice in Ctrl and UC groups, n = 6. Different lowercase letters indicate significant differences between groups.** (B-C)** Protein levels related to inflammatory cytokine in the colon of WT and SelS KO mice in Ctrl and UC groups, n = 3. Different lowercase letters indicate significant differences between groups.** (D-F)** MCECs were cocultured with J774.1 under IL-1β (100 ng/mL) stimulation.** (D)** mRNA expressions related to inflammatory cytokine in MCECs, n = 6. **(E-F)** Protein levels related to inflammatory cytokine in MCEC, n = 3. **(G-I)** MCECs were cocultured with VP (0.32 μM)-pretreated siNC or siSelS J774.1 under IL-1β (100 ng/mL) stimulation. **(G)** mRNA expressions related to inflammatory cytokine in MCECs, n = 6. **(H-I)** Protein levels related to inflammatory cytokine in MCECs, n = 3. **(J-L)** 1 mM NAC-pretreated MCECs were cocultured with siNC or siSelS J774.1 under IL-1β (100 ng/mL) stimulation. **(J)** mRNA expressions related to inflammatory cytokine in MCECs, n = 6.** (K-L)** Protein levels related to inflammatory cytokine in MCECs, n = 3.

Necroptosis is an inflammatory form of programmed cell death that plays a key role in driving inflammation initiation and aggravation. As shown earlier, SelS deficiency exacerbates UC colon injury primarily through CECs necroptosis. Therefore, the effect of SelS deletion on the inflammatory response of colon tissue was next analyzed. As shown in Figure 7A, markedly increased levels of several inflammatory factors, including TNF-α, IL-1β, IL-6, and IL-10 were observed in the WT mice of the UC group compared with the Ctrl group. The increased level of pro-inflammatory cytokines indicates the onset of an inflammatory response and the increase in IL-10 level may be compensatory to limit inflammatory exacerbation. Meanwhile, SelS KO mice exhibited an elevated level of pro-inflammatory cytokines and an extremely reduced level of IL-10, implying that the inflammatory response was amplified. However, IFN-γ and IL-17 levels were significantly increased only in SelS KO mice with UC and the protein expression of inflammatory factors showed the same trend as their mRNA expression (Figure 7B-C). *In vitro*, SelS knockdown increased the gene expression of TNF-α, IL-1β, and IL-6 and decreased gene expression of IL-10 in MCECs treated with IL-1β (Figure S10A-C), suggesting that SelS deficiency in CECs directly exacerbates intestinal inflammation in UC. The coculture of MCECs with IL-1β-induced J774.1 led to the upregulated gene expression of TNF-α, IL-1β, and IL-6 and downregulated gene expression of IL-10 in MCECs (Figure 7D-F). The SelS knockdown of J774.1 exacerbated the changing trend of the abovementioned inflammatory factor gene expression in MCECs (Figure 7G-I), corroborating that CECs necroptosis caused by SelS-silenced macrophages promotes inflammatory reaction. However, these effects were effectively eliminated by the blockade of YAP expression in J774.1 (Figure 7G-I) and scavenging of ROS in MCECs (Figure 7J-L). In summary, these results suggest that SelS deficiency induces M1 polarization by upregulating macrophage YAP expression, thereby promoting ROS-mediated necroptosis in CECs and inducing a cytokine storm.

### SelS ablation aggravates tight junction dysfunction depending on oxidative stress in CECs induced by M1 polarization

**Figure 8:**
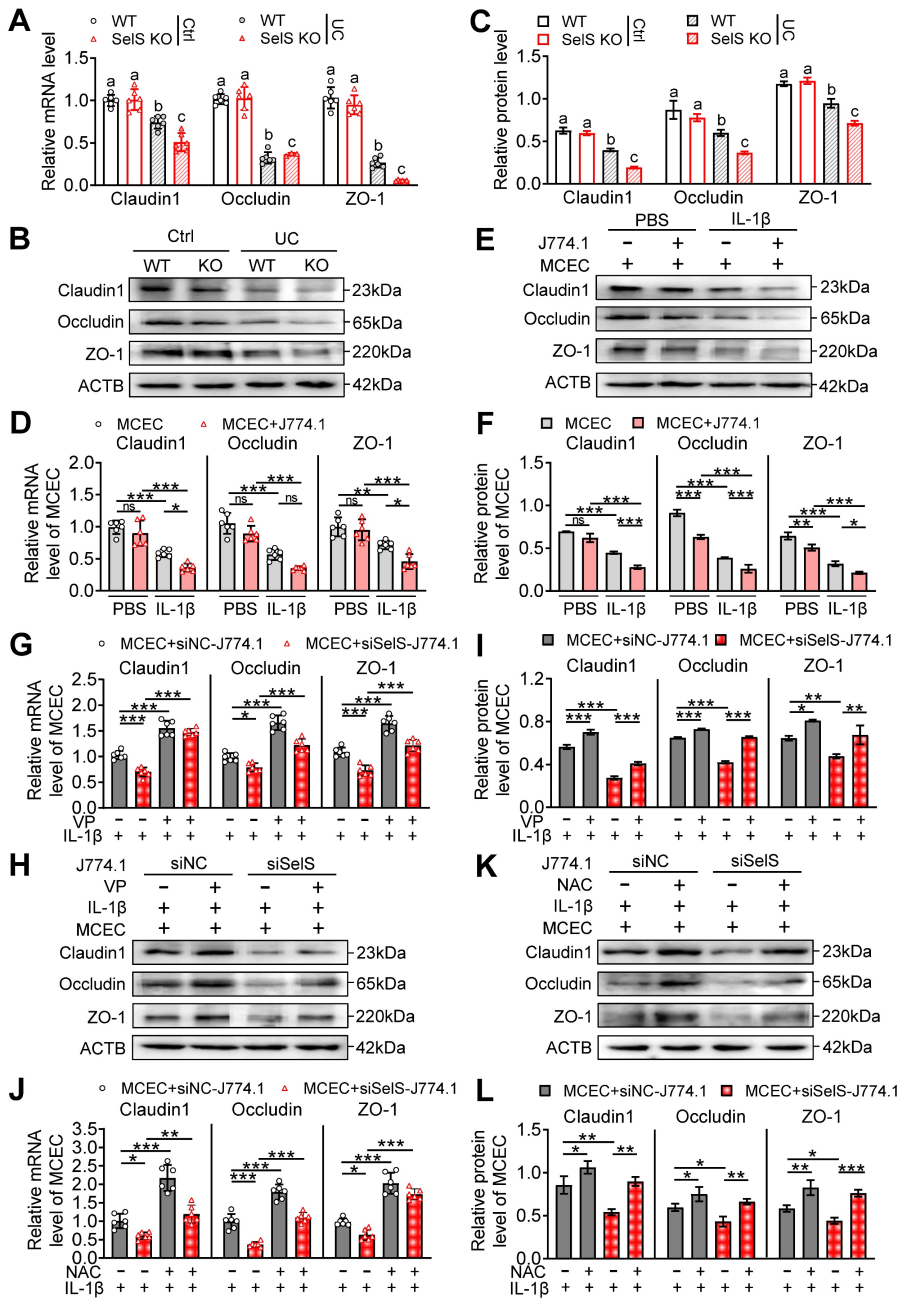
** SelS deletion aggravates tight junction dysfunction dependent on M1 polarization-induced oxidative stress in CECs. (A)** mRNA expressions related to tight junction in the colon of WT and SelS KO mice in Ctrl and UC groups, n = 6. Different lowercase letters indicate significant differences between groups.** (B-C)** Protein levels related to tight junction in the colon of WT and SelS KO mice in Ctrl and UC groups, n = 3. Different lowercase letters indicate significant differences between groups.** (D-F)** MCECs were cocultured with J774.1 under IL-1β (100 ng/mL) stimulation. **(D)** mRNA expressions related to tight junction in MCECs, n = 6. **(E-F)** Protein levels related to tight junction in MCECs, n = 3. **(G-I)** MCECs were cocultured with VP (0.32 μM)-pretreated siNC or siSelS J774.1 under IL-1β (100 ng/mL) stimulation. **(G)** mRNA expressions related to tight junction in MCECs, n = 6. **(H-I)** Protein levels related to tight junction in MCECs, n = 3. **(J-L)** 1 mM NAC-pretreated MCECs were cocultured with siNC or siSelS J774.1 under IL-1β (100 ng/mL) stimulation. **(J)** mRNA expressions related to tight junction in MCECs, n = 6.** (K-L)** Protein levels related to tight junction in MCECs, n = 3.

Gut barrier integrity is maintained by tight junction proteins. Necroptosis usually severely downregulates these proteins, leading to increased gut permeability to microbial ligands and noxious metabolites and ultimately to persistent and unresolved inflammation. To uncover the potential mechanisms of SelS in affecting tight junction proteins, the expression of tight junction-related genes in the colon tissue of WT mice and SelS KO mice was evaluated. As shown in Figure 8A-C, the mRNA and protein levels of Claudin1, Occludin, and ZO-1 in the UC group were significantly downregulated compared with the Ctrl group. Within the UC group, the above gene expression in SelS KO mice showed a greater downward trend than that in WT mice. Meanwhile, SelS knockdown downregulated the gene expression of Claudin1, Occludin, and ZO-1 in MCECs treated with IL-1β (Figure S10D-F). These results indicated that SelS deficiency in CECs directly exacerbates UC barrier damage. Meanwhile, the coculture of MCECs with J774.1 under IL-1β stimulation led to a reduction the above gene expression in MCECs (Figure 8D-F) and the expression of these tight junction genes in MCECs cocultured with siSelS J774.1 was lower than that in cells cocultured with siNC J774.1 (Figure 8G-I), indicating that macrophage SelS ablation triggers tight junction impairment in CECs. Next, to determine the role of macrophage polarization and oxidative stress in CECs in this process, J774.1 and MCECs were treated with VP and NAC, respectively. qRT-PCR revealed that the above interventions effectively inhibited the downregulation of tight junction-associated gene mRNA expression induced with SelS silencing (Figure 8G and J). In line with this, the expression pattern of the corresponding proteins was the same as that of the mRNA (Figure 8H-I and K-L). Taken together, these results suggest that the SelS ablation-induced impairment of tight junctions is mediated by exacerbating YAP-induced M1 polarization and ROS overproduction in CECs.

### Selenium supplementation ameliorates colon injury in UC

**Figure 9:**
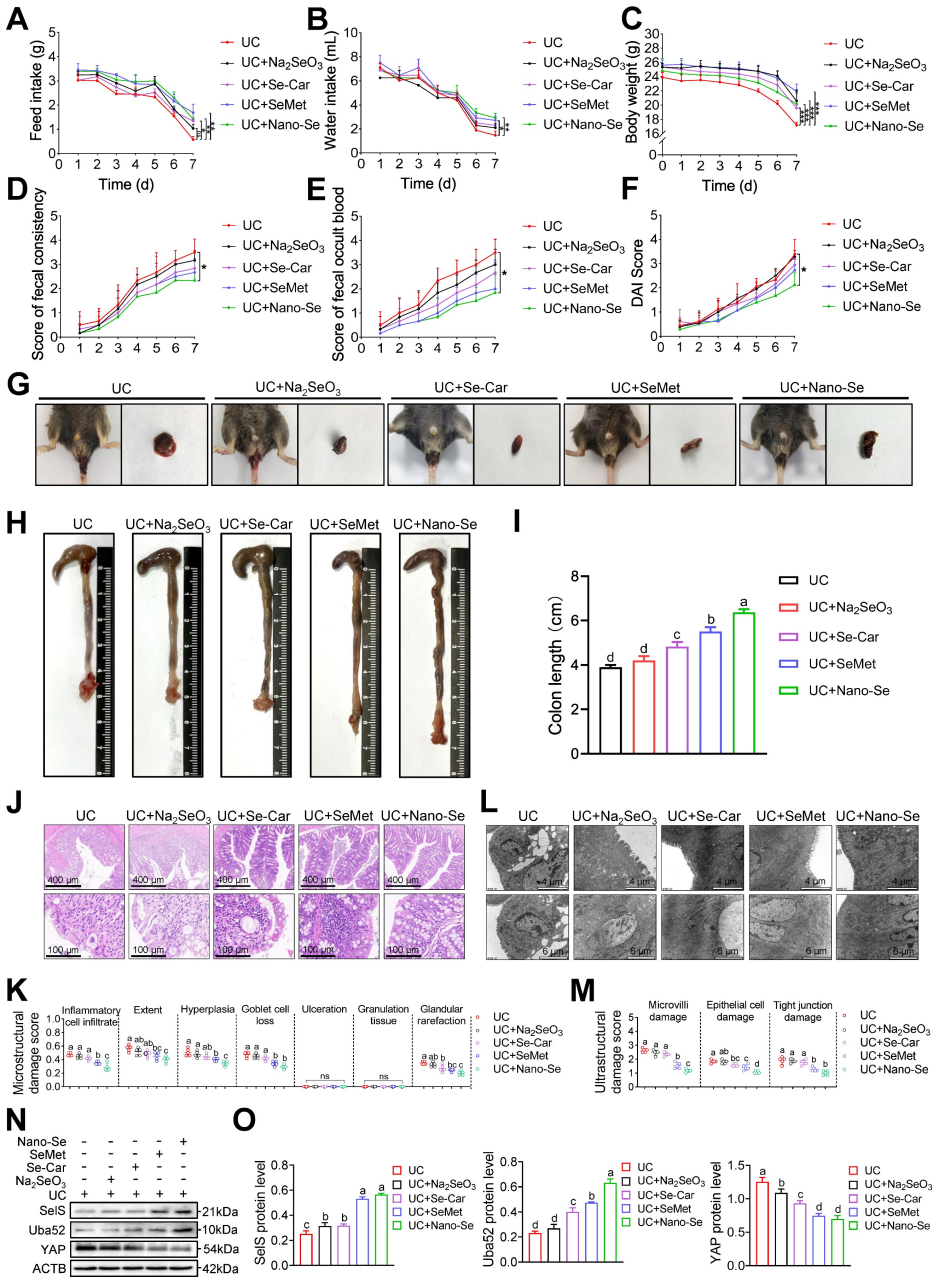
** Selenium supplementation attenuates colon injury in UC mice. (A-F)** Clinical indicators of WT mice with UC given selenium supplements containing 2 mg/kg selenium, n = 6. **(A)** Feed intake. **(B)** Water intake. **(C)** Body weight. **(D)** Scores of fecal consistency. **(E)** Scores of fecal occult blood. **(F)** DAI score. **(G)** Perianal area and feces appearance of UC mice. **(H-I)** Colon length, n = 6. Different lowercase letters indicate significant differences between groups. **(J)** Representative H&E slides of distal colon sections, n = 6. Scale bar, 400 μm and 100 μm. **(K)** The microstructural damage score of colon tissue, n = 6. Different lowercase letters indicate significant differences between groups. **(L)** Representative images of TEM detection for the colon tissue, n = 6. Scale bar, 4 μm and 6 μm. **(M)** The ultrastructural damage score of the colon tissue, n = 6. Different lowercase letters indicate significant differences between groups.** (N-O)** Protein levels of SelS, Uba52, and YAP in the colon, n = 3. Different lowercase letters indicate significant differences between groups.

As a critical trace element, selenium is considered as beneficial for the treatment of various intestinal diseases. To confirm whether selenium supplementation can attenuate colon injury in UC, UC mice were administered selenium in the form of four selenium preparations, including Na_2_SeO_3_, Se-Car, SeMet, and Nano-Se, via gavage. In general, selenium supplements increased feed intake, water intake, and body weight in UC mice (Figure 9A-C). In particular, Nano-Se significantly decreased fecal consistency, fecal occult blood, and DAI scores compared with UC mice (Figure 9D-G).

Likewise, colonic shortening (Figure 9H-I) as well as elevated microstructural (Figure 9J-K) and ultrastructural (Figure 9L-M) damage scores in UC mice were effectively ameliorated with selenium supplementation, with the most pronounced intervention effect observer with SeMet and Nano-Se. These results indicated that exogenous selenium supplementation helps improve the clinical manifestations of UC colon injury. Meanwhile, exogenous selenium supplementation is known to promote the induction of multiple selenoproteins. Therefore, whether selenium supplementation attenuates colon injury in UC by regulating SelS and its target proteins was next assessed. As shown in Figure 9N-O, compared with the UC group, the four selenium preparations increased the protein expression of SelS and Uba52 to varying degrees and decreased the protein expression of YAP, implying that selenium supplementation promotes SelS expression to upregulate Uba52, and subsequently suppresses of YAP expression.

The impact of selenium preparations on the macrophage polarization of the UC colon was next investigated. qRT-PCR revealed that the expression of the M1 markers iNOS, TNF-α, IL-6, IL-12, MCP-1, and MIG was significantly reduced after selenium supplementation in WT mice with UC (Figure S11A). By contrast, the expression of the M2 markers, CCL24, MRC1, Arg1, IL-4, IL-10, and Fizz1 was upregulated in selenium-supplemented mice (Figure S11B). These findings indicated an inadequate and enhanced expression of M1 and M2 markers, respectively, in the UC colon after selenium supplementation. The effect of selenium preparations on the redox state of the UC colon was also evaluated. As expected, selenium treatment reduced the MDA level and increased the GSH level and GPX, CAT, T-SOD, and T-AOC activities (Figure S11C). Additionally, the levels of proteins associated with the NF-κB/NLRP3 signaling pathway (Figure S11D-E), necroptosis (Figure S11F-G), and inflammatory cytokines (Figure S11H-I) was significantly reduced and the level of tight junction proteins (Figure S11J-K) was significantly increased in selenium-supplemented mice compared with UC mice. In the descender order, the ameliorative effect of selenium preparations on colon injury in UC mice was Nano-Se > SeMet > Se-Car > Na_2_SeO_3_. Taken together, these findings suggest that selenium supplementation ameliorates colon injury in UC by suppressing M1 macrophages, oxidative stress, necroptosis, inflammatory factor release, and tight junction damage. It appears that SelS targets Uba52 to regulate YAP in this process.

## Discussion

Patients with IBD usually experience malabsorption and micronutrient deficiencies 22, and selenium deficiency is a common manifestation in these patients 23, 24. Because it exerts its biological functions mainly through selenoproteins, the role of selenium and selenoproteins in UC has received extensive attention 13. In the present study, SelS deletion was shown to exacerbate inflammatory response and intestinal epithelial damage in UC. In addition, SelS knockdown in CECs led to ROS burst, necroptosis, inflammatory cytokine release, and tight junction disruption, whereas SelS silencing in macrophages promoted M1 polarization, which exacerbated ROS-dependent cascade damage in CECs and further worsened colon injury.

Growing evidence has suggested that some selenoproteins, such as thioredoxin reductase 3, SelP, and SelW, regulate intestinal immune homeostasis 25-27. SelS is a small type III single-pass transmembrane selenoprotein with a Sec residue near the C-terminus at position 188 28. He *et al.* demonstrated the protective anti-inflammatory effect of SelS using an siRNA knockdown strategy in a lipopolysaccharide-induced sepsis mouse model 29. *In vitro*, SelS gene expression was increased due to pro-inflammatory cytokines 30, which may be a protective strategy, as SelS knockdown increased the mRNA expression of pro-inflammatory cytokines in RAW264.7 macrophages 31. The current study results revealed that SelS expression in the CECs and colonic macrophages of UC mice showed an increasing trend. In addition, the DAI score, histopathological damage score, and inflammatory cytokine level in SelS KO mice with UC were increased, suggesting a protective role for SelS in intestinal inflammatory injury in UC. Pathological studies have confirmed the presence of significant immune cell infiltration in the diseased mucosal tissue of patients with UC, including macrophages 32. Besides, changes in macrophage polarization and related signaling pathways have been reported to have a vital impact on intestinal inflammation 33. YAP, a critical regulator of macrophage polarization, drives macrophages toward M1 polarization while restricting M2 polarization 34. Zhou *et al.* reported that YAP could bind to IL-6 promoter and enhance IL-6 production in bowel tissues 4. Liu *et al.* demonstrated that YAP binds directly to Arg1 promoter and inhibits Arg1 expression 34, 35. More interestingly, YAP expression was differentially regulated during the induction of macrophage polarization. Other studies demonstrated that IL-4/IL-13 treatment inhibited YAP expression while LPS/IFN-γ stimulation increased YAP protein expression in macrophages 36. In the present study, SelS deletion upregulated YAP expression, promoted M1 polarization, inhibited M2 polarization, and activated the NF-κB/NLRP3 signaling pathway in colon tissues and macrophages without exacerbating pyroptosis, in which the regulatory effects of other NLRP inflammasomes may be involved. Moreover, the alteration in macrophages was reversed with the YAP inhibitor VP, indicating that SelS affects macrophage polarization status via the negative regulation of YAP. The current dogma of YAP regulation is that the phosphorylation of YAP by upstream kinases in the Hippo signaling pathway is responsible for its ubiquitination and degradation 37. Surprisingly, the current study results revealed a novel mechanism of YAP regulation in a Hippo-independent manner by promoting YAP ubiquitination through Uba52-targeting SelS, thereby expanding the scope of YAP regulation. Additionally, GEO data mining analysis revealed that the expression level of SelS, Uba52, and YAP was higher in the colon of patients with UC than in normal subjects, which was consistent with the changes in gene expression detected in the colon of UC mice. Of note, these three genes were closely associated with multiple immune cell infiltration in patients with UC, including M1 and M2, which provided further support for the study findings.

It is widely recognized that heightened ROS production-mediated redox imbalance is associated with intestine damage. Clinical studies have determined increased total oxidative stress index and decreased antioxidant capacity in the plasma of patients with UC. The inflamed colonic mucosa of patients with IBD and animals with experimental colitis produces more ROS and less GSH than the normal colonic mucosa 38, 39. Ding *et al.* found that increased SelS expression inhibited intestinal oxidative stress in piglets 40. In the present study, the increase in pro-oxidant indicators and decrease in antioxidant enzyme activities were more pronounced in SelS-deficient colon tissues and CECs, demonstrating that SelS deficiency promotes oxidative stress in the CECs of UC. Hu *et al.* mimicked the microenvironment of enteritis by LPS treatment of cocultured RAW264.7 macrophages and intestinal epithelial-like Caco-2 cells and found a significant increase in cellular ROS levels 41. Moreover, ROS production was increased and antioxidant gene expression was downregulated in CECs cocultured with SelS-knockdown macrophages. Meanwhile. The VP and NAC treatment of macrophages and CECs, respectively, was effective in ameliorating the redox status of CECs because the two cell types did not come into direct contact but interacted through paracrine cytokines. These findings suggest that SelS deletion promotes the polarization of M1 by upregulating YAP and stimulates the production of more pro-inflammatory cytokines that act on CECs, thereby increasing the level of oxidative stress in the latter.

Research has shown that diffuse inflammatory cell infiltration and small intestinal mucosal crypt abscesses in colitis promote excessive ROS production, leading to oxidative stress damage in colonocytes, increased epithelial barrier permeability, and pathogen invasion while exacerbating inflammatory cell infiltration and inflammatory injury 42. Studies have shown that intracellular ROS accumulation in response to external inflammatory substances triggers necroptosis 7, which induces an inflammatory response in IECs and alters their cell membrane permeability 43. Recent studies have shown that RIPK3 expression in the colon is positively correlated with the severity of UC 44. Likewise, Pierdomenico *et al.* found that the expression level of RIPK3 and MLKL was significantly increased in the colon tissues of children with UC and CD and that the expression level of caspase-8 was markedly decreased, which is consistent with the fact that necroptosis occurs independent of caspase-8 but is dependent on RIPK3 and MLKL regulation 45. Of note, the necroptosis inhibitor Nec-1 improved intestinal histopathology in DSS-induced colitis mice 44. Other studies showed that SelS is unable to protect IEC from oxidative stress-induced apoptosis 17. The present study indicated that SelS deficiency aggravated UC colon injury by inducing CEC necroptosis, independent of cell proliferation, apoptosis, and pyroptosis, thereby uncovering new evidence for the function of SelS in IECs. Li *et al.* studied the regulatory effect of SelS on necroptosis and demonstrated that SelS knockdown decreased mitochondrial membrane potential and ATP depletion through increased ROS production, which, in turn, transformed apoptosis into necroptosis 46. Additionally, ROS produced by macrophages or other immune cells directly damaged CECs during UC 47. The present study results revealed that in a coculture system of SelS-silenced macrophages and CECs, the inhibition of YAP expression in macrophages and ROS production in CECs reduced the number of necrotic cells and expression of necroptosis-related genes while decreasing the gene expression pro-inflammatory cytokines and increasing the gene expression of anti-inflammatory cytokines and tight junctions. Therefore, SelS deficiency in macrophage promotes YAP-mediated M1 polarization, thereby aggravating necroptosis, inflammatory factor release, and tight junction impairment in CECs via ROS overproduction.

Serum selenium levels have been shown to be significantly lower in patients with quiescent UC 48. Data from animal studies suggest that adequate dietary selenium reduces intestinal inflammation 49. Indeed, the selenium status affects gene expression, signaling pathways, and cellular functions in the gut. Related studies have proposed that several selenoproteins may be involved in selenium-mediated protection against intestinal inflammation, including GPX isozymes, SelS, and SelP, and that all of these selenoproteins may have immunomodulatory functions 12. Barger *et al.* demonstrated that feeding selenium to mice in the form of Na_2_SeO_3_, selenium-enriched yeast, and SeMet at a concentration of 1 mg/kg selenium induced a sustained upregulation of GPX1 and SelW 50. The present study results showed that supplementation with different selenium sources containing 2 mg/kg selenium effectively increased the protein level of SelS, accompanied by an upregulation of Uba52 and downregulation of YAP. Other studies have confirmed that selenium supplementation attenuated DSS-induced experimental colitis in WT mice. However, selenium supplementation did not protect against DSS-induced colitis in mice that lacked selenoprotein expression in macrophages 15, suggesting that selenoprotein expression in macrophages is critical for the protective role of selenium in colitis. Besides, a study showed that supplementation with a supra-nutritional dose of selenite (0.4 mg/mL) upregulated the expression of M2 markers and concomitantly downregulated the expression of M1 markers in the colon tissue of UC mice treated with the DSS 15. The current study revealed similar effects on M1 and M2 markers in the colon tissue of UC mice supplementation with 2 mg/kg selenium. Moreover, selenium supplementation reduced oxidative stress, inhibited necroptosis, decreased inflammatory cytokine expression, and enhanced tight junction repair in the colon of UC mice. Of note, a previous study revealed that GPX4 and SelS synergistically regulated oxidative stress-induced IEC damage and that SelS exhibited a stronger regulatory effect 40. Therefore, the regulatory effect of exogenous selenium supplementation on UC may involve the interaction among multiple selenoproteins, which deserves further exploration. Zhong *et al.* demonstrated that organic selenium had a stronger modulatory effect on both inflammatory cytokines and tight junction proteins in the colon tissue of UC mice than Na_2_SeO_3_ 51. The present study demonstrated that the mitigating effect of different forms of selenium on colon injury in UC mice varied greatly, with the extent of effect being Nano-Se > SeMet > Se-Car > Na_2_SeO_3_, which may be attributed to their different catabolic pathways and utilization of selenoprotein biosynthesis.

## Conclusion

In summary, selenium is an essential micronutrient uniquely incorporated into various selenoproteins to confer beneficial functions. In addition, SelS deficiency not only directly induces oxidative stress, necroptosis, inflammatory factor release, and tight junction injury in CECs but also enhances CEC injury by promoting YAP-mediated M1 polarization via Uba52 downregulation in macrophages, which ultimately aggravates colon injury in UC. Meanwhile, selenium supplementation, which upregulated SelS expression to some extent, may be a possible strategy to alleviate macrophage polarization and mitigate CEC injury in UC. Of note, the effect of Nano-Se was the strongest among the selenium supplements. Taken together, the study results elucidated the mechanism through which SelS regulates the immunity of UC colonic mucosa and provided an important therapeutic target to improve the inflammatory response and epithelial damage in UC colon tissues.

## Supplementary Material

Supplementary figures and table.
