# Supplementary material for: Selenoprotein S maintains intestinal homeostasis in ulcerative colitis by inhibiting necroptosis of colonic epithelial cells through modulation of macrophage polarization
Source: Theranostics. 2024 Sep 9;14(15):5903–25. doi: 10.7150/thno.97005 (PMC11426251; doi:10.7150/thno.97005)
Supplement: Supplementary file 1 — Supplementary figures and table. [file thnov14p5903s1.pdf]

*Supplemental Materials*

# Selenoprotein S maintains intestinal homeostasis in ulcerative colitis by inhibiting necroptosis of colonic epithelial cells through modulation of macrophage polarization

Yujie Yao[a, b], Tong Xu[a], Xiaojing Li[c], Xu Shi[a], Hao Wu[a], Ziwei Zhang[a, d, *], Shiwen Xu[a, d, *]

*a. College of Veterinary Medicine, Northeast Agricultural University, Harbin, 150030, PR China*

*b. School of Tropical Agriculture and Forestry, Hainan University, Haikou, 570228, PR China*

*c. College of Animal Science and Technology, Northeast Agricultural University, Harbin, 150030, PR China*

*d. Key Laboratory of the Provincial Education Department of Heilongjiang for Common Animal Disease Prevention and Treatment, College of Veterinary Medicine, Northeast Agricultural University, Harbin, 150030, PR China*

* Corresponding author

*College of Veterinary Medicine, Northeast Agricultural University, Harbin, 150030, PR China*

*E-mail address:* zhangziwei@neau.edu.cn (Z. Zhang), shiwenxu@neau.edu.cn (S. Xu).

Contributor information:

Yujie Yao, Email: yujieyao@hainanu.edu.cn

Tong Xu, Email: 13147661321@163.com

Xiaojing Li, Email: xiaojingli@neau.edu.cn

Xu Shi, Email: 694478106@qq.com

Hao Wu, Email: S200601044@neau.edu.cn

Ziwei Zhang, Email: zhangziwei@neau.edu.cn

Shiwen Xu, Email: shiwenxu@neau.edu.cn

## Table S1. Sequences of oligonucleotide primers for qRT-PCR

| Gene Names | Forward Primer (5'→3') | Reverse Primer (5'→3') |
|---|---|---|
| GPX1 | AGTCCACCGTGTATGCCTTC | GTGTCCGAACTGATTGCACG |
| GPX2 | CCTACCGGCCATTTCCTTTA | GACATCTCCCAGAAGGGTTTAG |
| GPX3 | CCCTTAGTGCATTCAGGCTTAG | GATACCAGTGGACAGAGTGAGA |
| GPX4 | GCCGTCTGAGCCGCTTACTTA | CGTCGATGTCCTTGGCTGAG |
| Txnrd1 | ACAGCGAGGAGACCATAGA | CCACGGTCTCTAAGCCAATAG |
| Txnrd2 | CATCTTCTGGCTGAAGGAGTC | ACAGTGGTATCCAGTCCAATTC |
| Txnrd3 | GAAGGACCGGAAACAGTAGAA | CGATCTTCTCCAGCCCTATTT |
| Dio1 | TGGTTTGTCCTGAAGGTCCG | GTCAGGTGCAACTGCCAAAG |
| Dio2 | TCCTACAGTCACAATGCTACAC | GGTTAGTGTACCTGCCTCTTAC |
| Dio3 | CTCGAAACAGCGCCTAAAGTA | GAAGTCCATCCCTTACCATGTC |
| SelN | ATGATCTGTCTGCCCAACGG | GTCCTTCCCTCAGGAACTGC |
| SelK | GTGCAGGAGAAGATGGTGTATG | TGCGTATCAGTCAGGCTAGA |
| SelW | GCCGTTCGAGTCGTGTATTG | GCCACAAATGTCCAGGCATC |
| SelT | GAAGTTATGAGCTGAGGGAAGG | CAGTGTGTGTCCAGTAGTCAAG |
| SelH | GAGCATTGTACGAGCTGACG | AGTCCAGAGTTCAACACGGC |
| SelM | CCACCACCAACTACCGACC | GGCCTTCACCTCCTTTAGGC |
| SelF | GAAGTAGCACCACAGTCCATAA | GGTGAGAGCAACAAGTGTAGAA |
| SelI | GGAGTGTTCTGCCGTTACTT | GAGGAACCGCCACAATTAGA |
| SelV | GCTATGGCCTTCGGTACATTAT | CTGTAACCTGGGTAGCTCTTTC |
| SelP | GCCAATAAAACTGCGGAGCC | GCAAGCCTTCACTTGCTGTG |
| SelO | CACTATCGACTATGGACCCTTTG | GGGCTGCTTACTGTATGTGTAG |
| SelR | AGTTCGTCCCTAAAGGCAAAG | AGGTTCTGACACTAACGTGGG |
| SelS | CTTTGCGAGGAGGTGGTTAT | GTCAGAGCGACACTAACAAGAG |
| Sephs2 | CCCACCAATGGCTGGATAAT | GCAGCAGTCCTGTTTAGAGTAG |
| MIG | AACGTTGTCCACCTCCCTTC | CACAGGCTTTGGCTAGTCGT |
| TNF-α | CCTGTAGCCCACGTCGTAGC | AGCAATGACTCCAAAGTAGACC |
| iNOS | CAACAGGGAGAAAGCGCAA | CTCACATACTGTGGACGGGT |
| IL-6 | ATCCAGTTGCCTTCTTGGGACTGA | TTGGATGGTCTTGGTCCTTAGCCA |
| IL-12 | GGAAGCACGGCAGCAGAATA | AACTTGAGGGAGAAGTAGGAATGG |
| MCP1 | CTCACCTGCTGCTACTCATTC | TTACGGCTCAACTTCACATTCA |
| Arg1 | TGTGAAGAACCCACGGTCTG | ACGTCTCGCAAGCCAATGTA |
| Fizz-1 | AACTGCCTGTGCTTACTCGT | CAAGAAGCAGGGTAAATGGGC |
| IL-4 | ACTTGAGAGAGATCATCGGCATTT | AGCACCTTGGAAGCCCTACAG |
| IL-10 | ACTTGGGTTGCCAAGCCTTA | GACACCTTGGTCTTGGAGCTTA |
| MRC1 | GTCAGAACAGACTGCGTGGA | AGGGATCGCCTGTTTTCCAG |
| CCL24 | CCCTGAACTTGGACATAGGGG | AAAATGCTGCTGTTGAAATCCT |

| Gene Names | Forward Primer (5’→3’) | Reverse Primer (5’→3’) |
| --- | --- | --- |
| Uba52 | TTTCTCTTCAACGAGGCGGC | GGTATGGCCGCACTTCTTCT |
| YAP | CCCTCGTTTTGCCATGAACC | TCCGTATTGCCTGCCGAAAT |
| MST1 | AGACCCGAATGTTGAGCGAG | TTTCAAACACCACCTGGTCTGG |
| LATS1 | CGTCCCCTGCAGGTTAGTTA | AGGTCTTCCTACATCCGCCT |
| NF-κB-p105 | GGTCAAAATTTGCAACTATGTGGG | GCGTGCAGGTGGATGTTTTT |
| NLRP3 | CAAGGCTGCTATCTGGAGGAAC | TCGCAGCAAAGATCCACACA |
| ASC | CCATCCTGGACGCTCTTGAA | GTGAGCTCCAAGCCATACGA |
| Caspase1 | ACTGCTATGGACAAGGCACG | GCAAGACGTGTACGAGTGGT |
| GSDMD | ATCAAGGAGGTAAGCGGCAG | CCTTCTCCCATGCCTGACAA |
| IL-1β | TGGACCTTCCAGGATGAGGACA | GTTCATCTCGGAGCCTGTAGTG |
| IL-18 | GACAGCCTGTGTTCGAGGATATG | TGTTCTTACAGGAGAGGGTAGAC |
| CAT | TCACTGACGAGATGGCACAC | ATCGAACGGCAATAGGGGTC |
| GST | GAACTATACAGGTCGGCGATG | AGCTTGGGATGAAAGACTCCG |
| SOD1 | GCTTCTCGTCTTGCTCTCTCTG | TCTGCTCGAAGTGGATGGTT |
| SOD2 | TAACGCGCAGATCATGCAGCTG | AGGCTGAAGAGCGACCTGAGTT |
| FADD | CCCCTCAGCACACTTGAACT | GTGACTTGCCACAGGACAGT |
| RIPK1 | GGCCATAGAAGGCATGTGCT | GCCACACCAAGATCGGCTAT |
| RIPK3 | GGCTGGCACTCCTCAGATTC | GTGCCGTGTCTTCCATCTCC |
| MLKL | CCGGACAGCAAAGAGCACTA | CTGCCAGAAAGACTCCTACCG |
| Claudin1 | TTTGGCCAGGCCCTCTTTAC | GGAGCACCTTATCCCCGTTT |
| Occludin | GAGTTTCAGGTGAATGGGTCAC | AGGCAAATATGGCGATGCAC |
| ZO-1 | TTCCGGGGAAGTTACGTGC | CCATTGCTGTGCTCTTAGCG |
| ACTB | CCTCTATGCCAACACAGTGC | CCTGCTTGCTGATCCACATC |

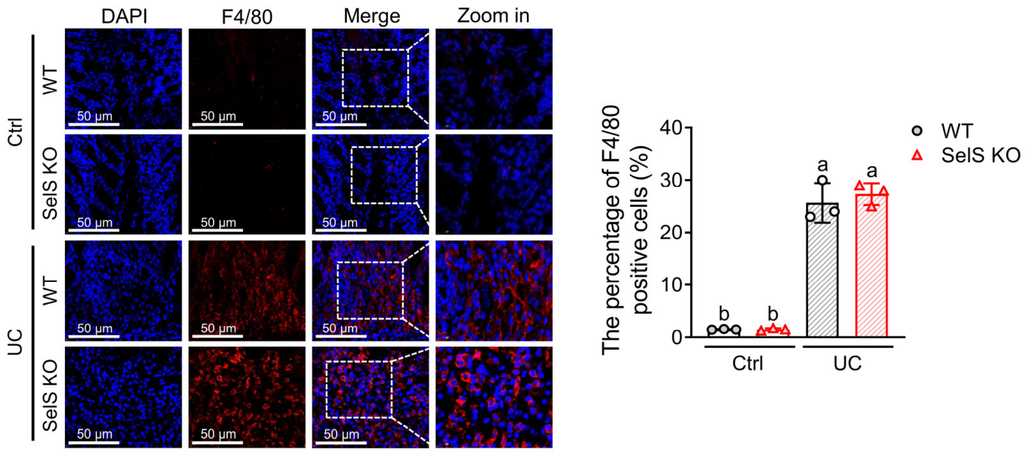


**Figure S1. SelS deletion enhances inflammatory response of the colon tissue in UC mice independent of macrophage infiltration.** Immunofluorescence staining and quantification analysis of F4/80 in the colon tissue of WT and SelS KO mice in Ctrl and UC groups, n = 3. Scale bar, 50 μm.

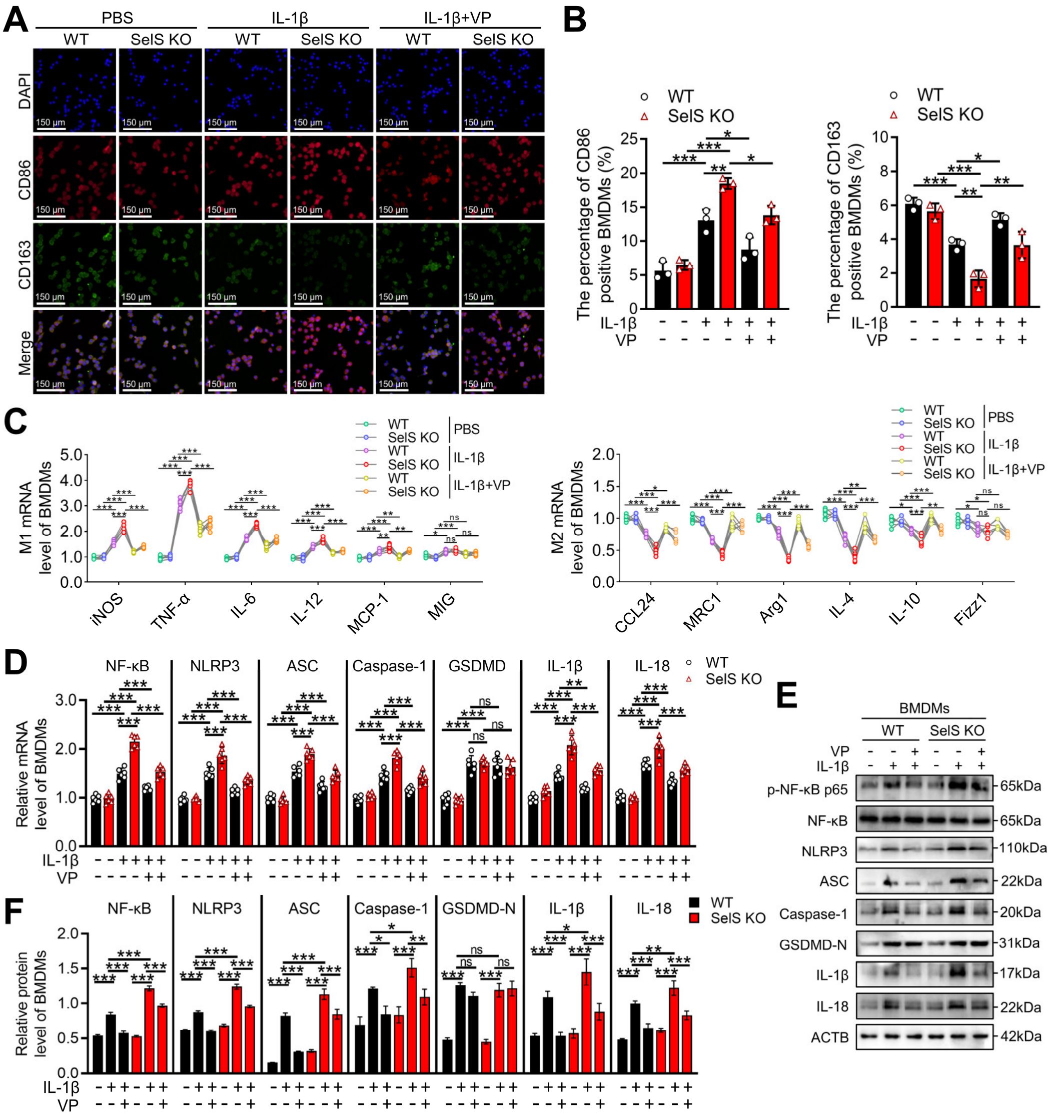


**Figure S2. SelS deficiency in BMDMs promotes YAP-mediated M1 polarization and NF-κB/NLRP3 pathway activation. (A-E)** BMDMs transfected with siNC or siSelS were pretreated with or without VP (0.32 μM) for 30 min before PBS or IL-1β (100 ng/mL) stimulation for 6 h. **(A)** Immunofluorescence staining and quantitative analysis of CD86 and CD163 in BMDMs, n = 3. Scale bar, 150 μm. **(B)** M1 and M2 related mRNA expressions in BMDMs, n = 6. **(C)** mRNA expressions related to the NF-κB/NLRP3 pathway and GSDMD in BMDMs, n = 6. **(D-E)** Protein levels related to the NF-κB/NLRP3 pathway and GSDMD in BMDMs, n = 3.

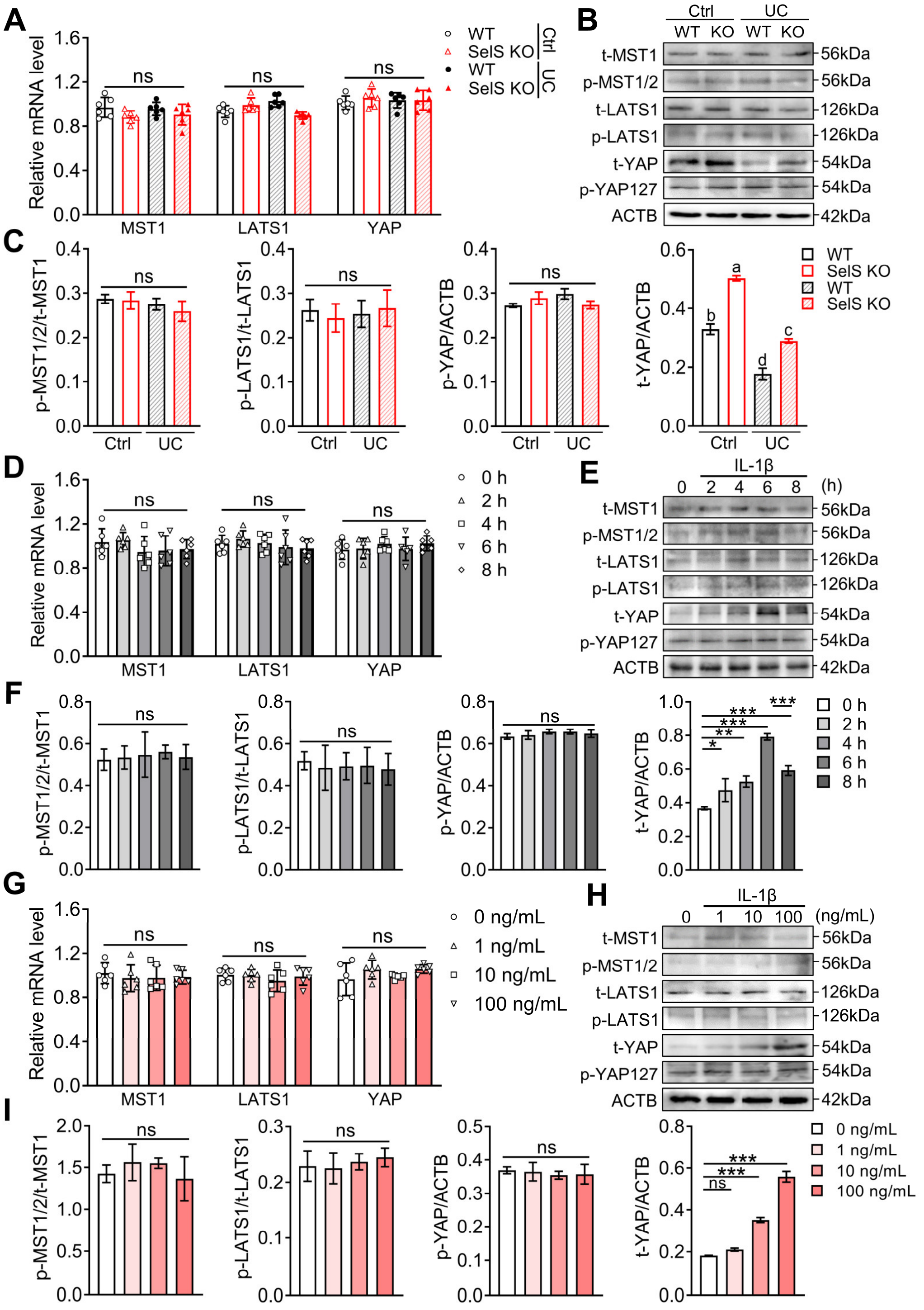


**Figure S3. IL-1β increases YAP protein level independent of the traditional Hippo pathway. (A)** mRNA expressions of MST1, LATS1, and YAP in the colon tissue of WT and SelS KO mice in Ctrl and UC groups, n = 6. **(B-C)** Protein levels of MST1/2, LATS1, and YAP in the colon tissue of WT and SelS KO mice in Ctrl and UC groups. t, total; p, phosphorylated, n = 3. **(D-F)** J774.1 were treated with IL-1β (100 ng/mL) for the indicated times. **(D)** mRNA expressions of MST1, LATS1, and YAP in J774.1, n = 6. **(E-F)** Protein levels of MST1, LATS1, and YAP in J774.1, n = 3. **(G-I)** J774.1 were stimulated with indicated concentrations of IL-1β for 6 h. **(G)** mRNA expressions of MST1, LATS1, and YAP in J774.1, n = 6. **(H-I)** Protein levels of MST1, LATS1, and YAP in J774.1, n = 3.

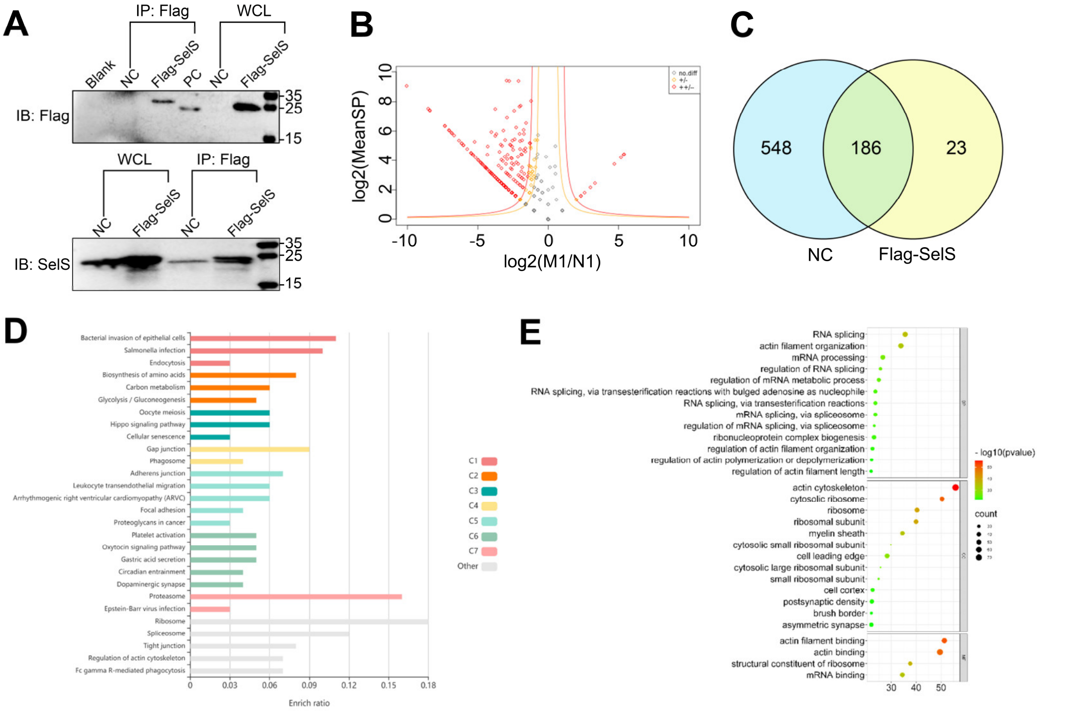


**Figure S4. CoIP-MS screens the interacting proteins of SelS in Hepa1-6. (A)** The IB of Flag or SelS was followed by IP with anti-Flag antibody in WCL, n = 3. Blank, blank control; NC, transfection of empty plasmids; Flag-SelS, transfection of Flag-SelS overexpression plasmid; PC, positive control; WLC, whole-cell lysates. **(B)** Analysis of differentially expressed proteins in IP by mass spectrometry. **(C)** Venn diagram showing the protein composition in IP of NC group and Flag-SelS group. **(D)** KEGG pathway enrichment of differential proteins in IP of NC group and Flag-SelS group. **(E)** GO function annotation of differential proteins in IP of NC group and Flag-SelS group.

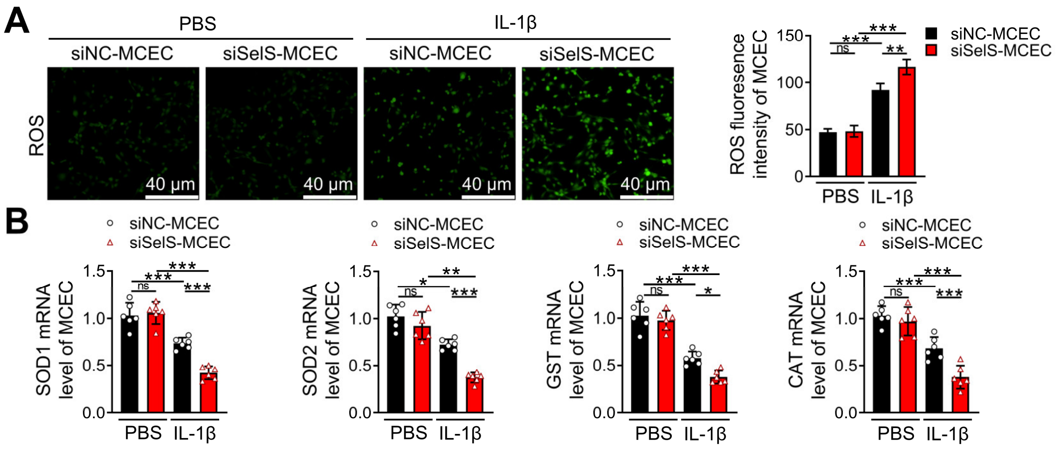


**Figure S5. SelS deficiency in MCECs promotes oxidative stress. (A)** Representative images of ROS levels in siNC and siSelS MCECs under IL-1β (100 ng/mL) stimulation, n = 3. Scale bar, 40 μm. **(B)** mRNA expressions of antioxidase in siNC and siSelS MCECs under IL-1β (100 ng/mL) stimulation, n = 6.

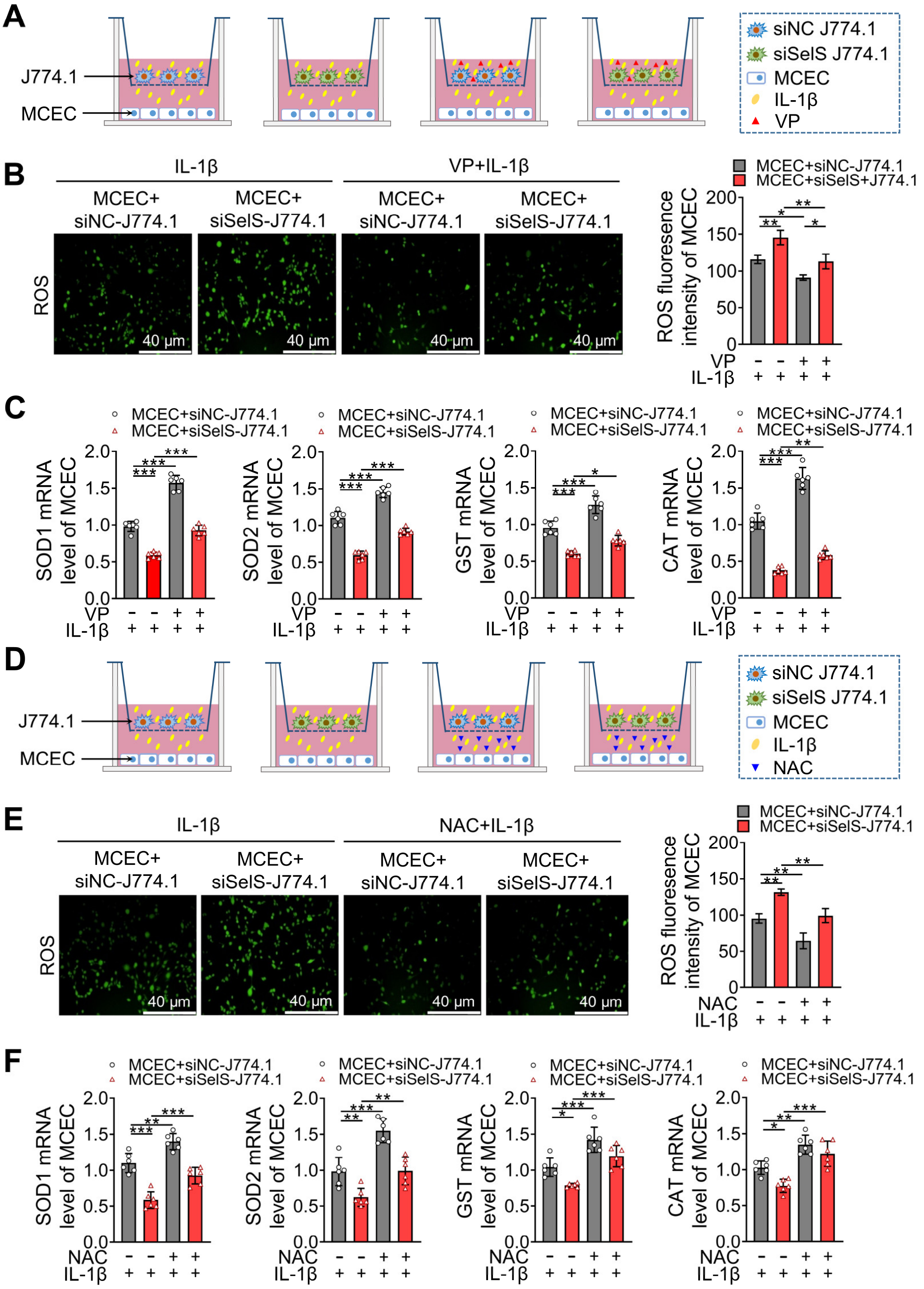


**Figure S6. SelS deficiency in J774.1 increases oxidative stress in MCECs by promoting YAP expression. (A-C)** MCECs were co-cultured with 0.32 μM VP-pretreated siNC or siSelS J774.1 under IL-1β (100 ng/mL) stimulation. **(A)** Co-culture pattern diagram of MCECs and J774.1. **(B)** Representative images of ROS levels in MCECs, n = 3. Scale bar, 40 μm. **(C)** mRNA expressions of antioxidase in MCECs, n = 6. **(D-F)** 1 mM NAC-pretreated MCECs were co-cultured with siNC or siSelS J774.1 under IL-1β (100 ng/mL) stimulation. **(D)** Co-culture pattern diagram of MCECs and J774.1. **(E)** Representative images of ROS levels in MCECs, n = 3. Scale bar, 40 μm. **(F)** mRNA expressions of antioxidase in MCECs, n = 6.

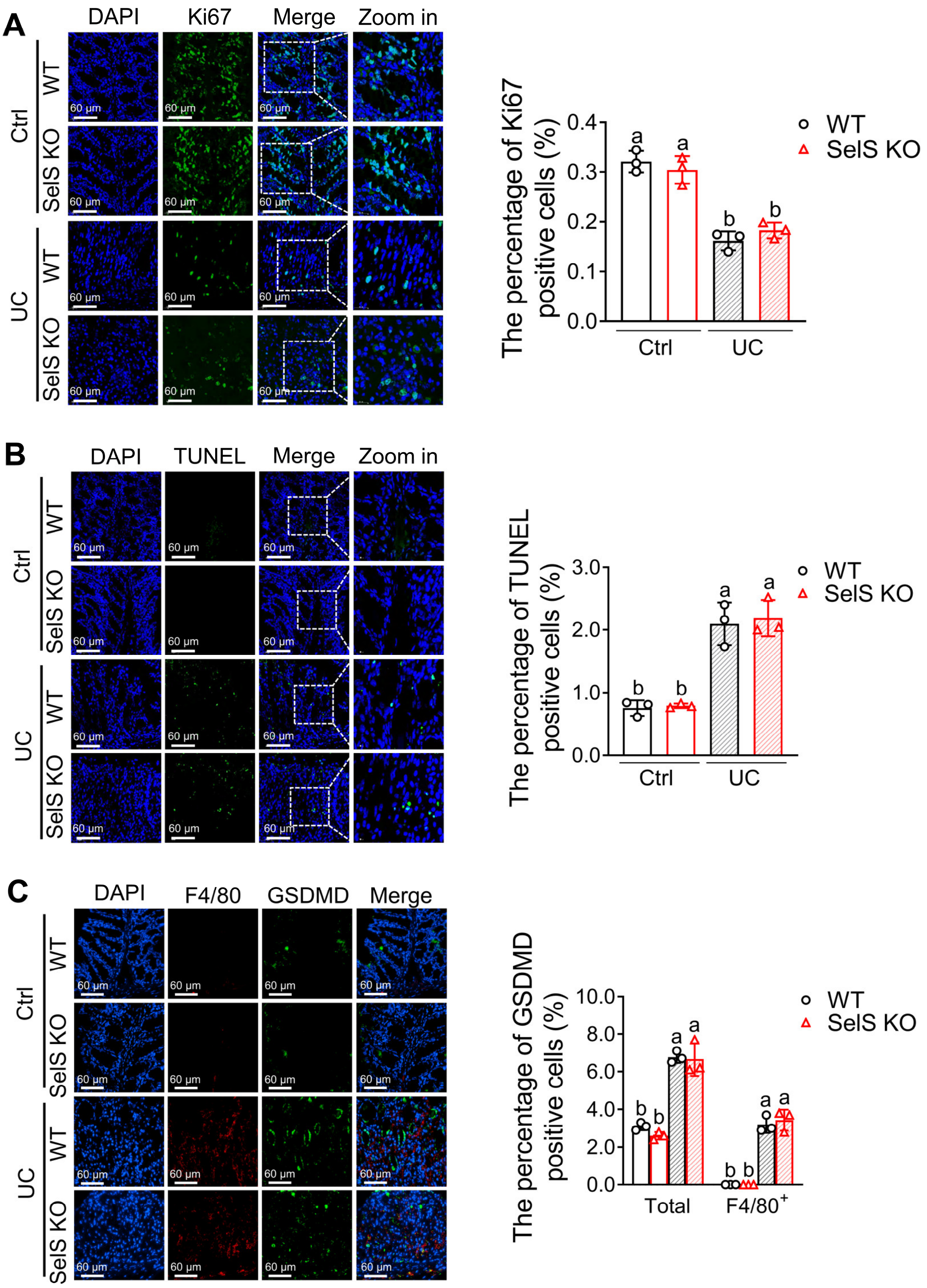


**Figure S7. SelS deletion promotes colon injury in UC mice independent of cell proliferation, apoptosis, and pyroptosis. (A)** Immunofluorescence staining and quantitative analysis of Ki67 in the colon tissue of WT and SelS KO mice in Ctrl and UC groups, n = 3. Scale bar, 60 μm. **(B)** TUNEL staining and quantitative analysis in the colon tissue of WT and SelS KO mice in Ctrl and UC groups, n = 3. Scale bar, 60 μm. **(C)** Immunofluorescence staining and quantitative analysis of F4/80 and GSDMD in the colon tissue of WT and SelS KO mice in Ctrl and UC groups, n = 3. Scale bar, 60 μm.

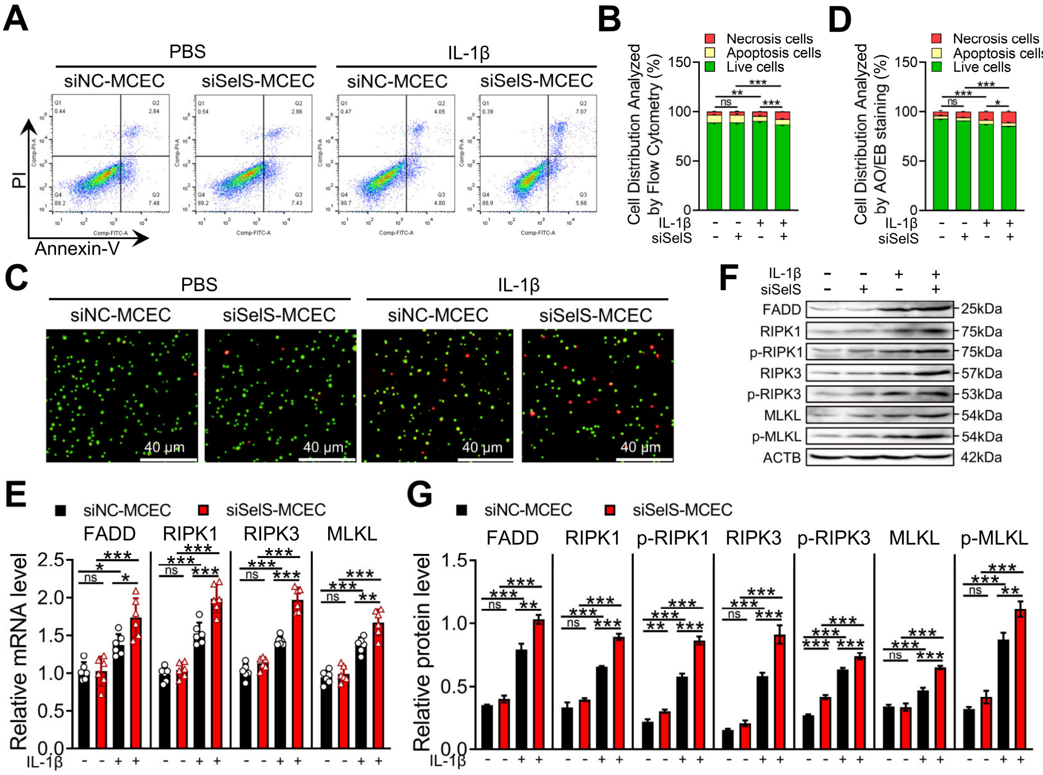


**Figure S8. SelS deficiency in MCECs promotes necroptosis. (A-G)** siNC and siSelS MCECs were treated with IL-1β (100 ng/mL). **(A-B)** Flow cytometry detection and quantification analysis for FITC/PI staining in MCECs, n = 3. **(C-D)** AO/EB staining and quantification analysis in MCECs, n = 3. Scale bar, 40 μm. **(E)** mRNA expressions related to necroptosis in MCEC, n = 6. **(F-G)** Protein levels related to necroptosis in MCECs, n = 3.

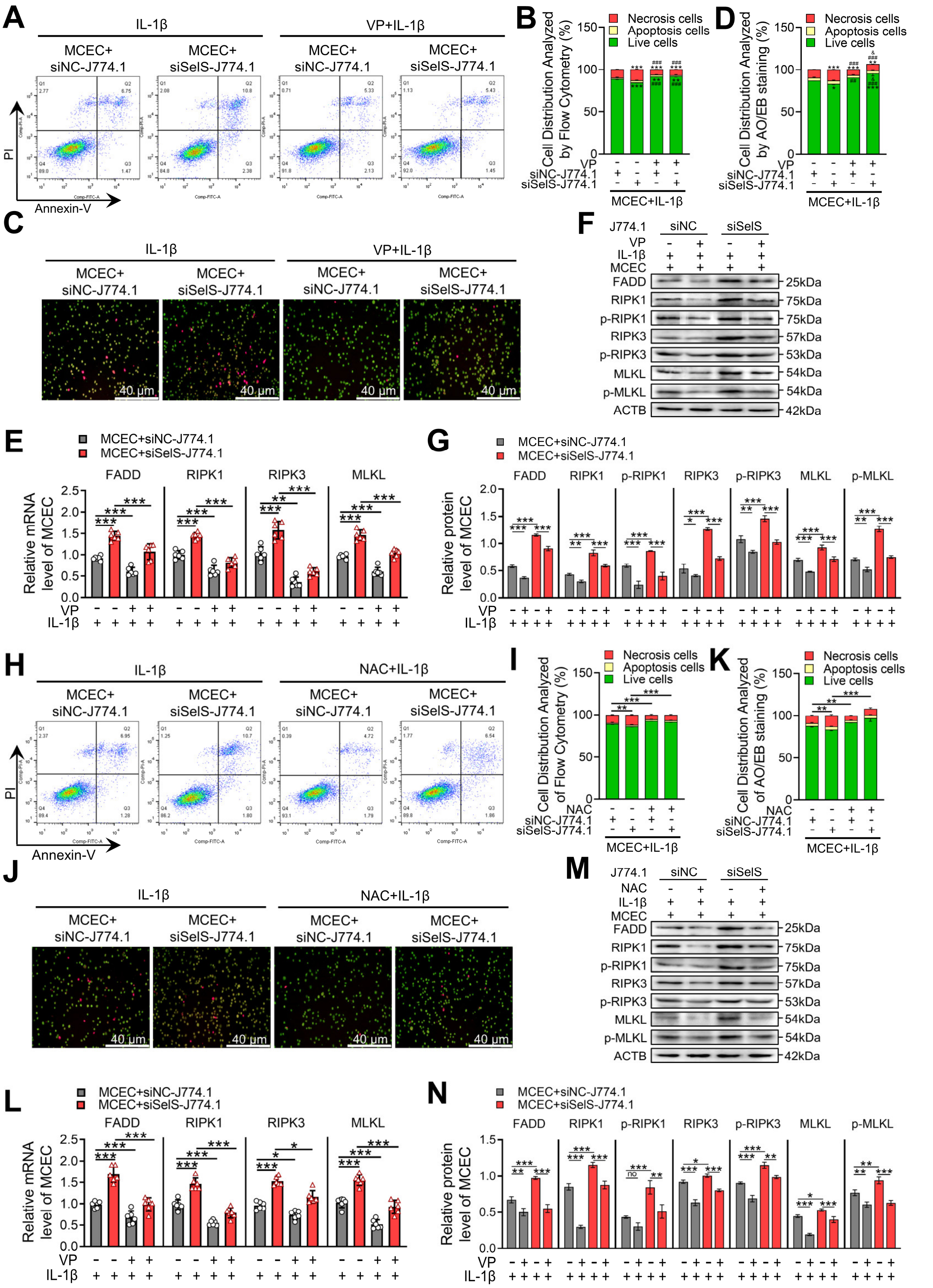


**Figure S9. SelS deficiency in J774.1 promotes YAP expression to induce ROS-dependent necroptosis in MCECs. (A-G)** MCECs were co-cultured with 0.32 μM VP-pretreated siNC or siSelS J774.1 under IL-1β (100 ng/mL) stimulation. **(A-B)** Flow cytometry detection and quantification analysis for FITC/PI staining in MCECs, n = 3. **(C-D)** AO/EB staining and quantification analysis in MCECs, n = 3. Scale bar, 40 μm. **(E)** mRNA expressions related to necroptosis in MCECs, n = 6. **(F-G)** Protein levels related to necroptosis in MCECs, n = 3. **(H-N)** 1 mM NAC-pretreated MCECs were co-cultured with siNC or siSelS J774.1 under IL-1β (100 ng/mL) stimulation. **(H-I)** Flow cytometry detection and quantification analysis for FITC/PI

staining in MCECs, n = 3. **(J-K)** AO/EB staining and quantification analysis in MCECs, n = 3. Scale bar, 40 μm. **(L)** mRNA expressions related to necroptosis in MCECs, n = 6. **(M-N)** Protein levels related to necroptosis in MCECs, n = 3.

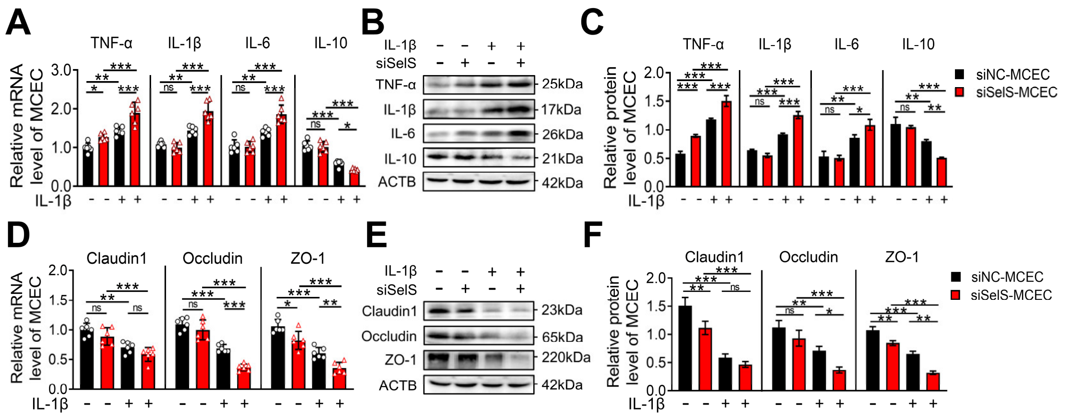


**Figure S10. SelS deficiency in MCECs promotes inflammatory response and tight junction impairment. (A-G)** siNC and siSelS MCECs were treated with IL-1β (100 ng/mL). **(A)** mRNA expressions related to inflammatory cytokines in MCECs, n = 6. **(B-C)** Protein levels related to inflammatory cytokines in MCECs, n = 3. **(D)** mRNA expressions related to tight junction in MCECs, n = 6. **(E-F)** Protein levels related to tight junction in MCECs, n = 3.

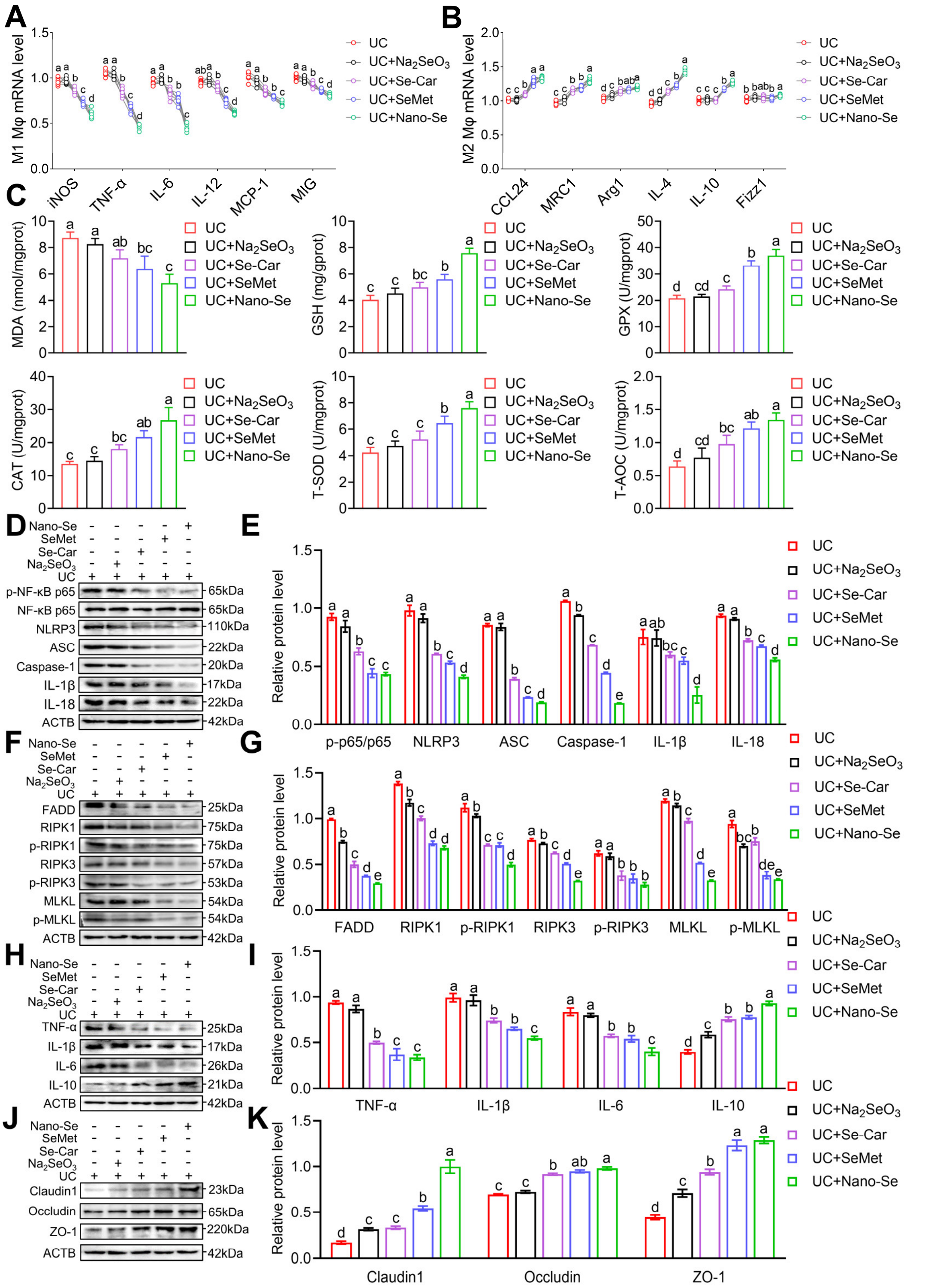


**Figure S11. Selenium supplementation reverses macrophage polarization, oxidative stress, necroptosis, inflammatory response, and tight junctions in the colon tissue of UC mice. (A-G)** WT mice with UC were given selenium supplements containing 2 mg/kg selenium. **(A-B)** mRNA expressions related to M1 and M2 in the colon tissue, n = 6. **(C)** Levels of pro-oxidant indicators and antioxidant markers in the colon tissue, n = 6. **(D-E)** Protein levels related to the NF-κB/NLRP3 pathway in the colon tissue, n = 3. **(F-G)** Protein levels related to necroptosis in the colon tissue, n = 3. **(H-I)** Protein levels related to inflammatory cytokine in the colon tissue, n = 3. **(J-K)** Protein levels related to tight junction in the colon tissue, n = 3.
